# Exercise-derived exosomal miR-151-3p: An innovative anti-inflammatory and antioxidant therapeutic for spinal cord injury

**DOI:** 10.1016/j.bioactmat.2026.06.009

**Published:** 2026-06-13

**Authors:** Xinwang Ying, Jiamen Shen, Yanfang Zhao, Tiantaixi Tu, Yi Jiang, Bo Chen, Zhiyang Huang, Qingfeng Xie, Liuxi Chu, Junqing Huang, Yanming Zuo, Ao Fang, Ping Wu, Qian Xu, Xiaokun Li, Chang Jiang, Zhouguang Wang

**Affiliations:** a The First Affiliated Hospital of Wenzhou Medical University, Wenzhou, Zhejiang, 325035, China; b The Orthopaedic Center, The Affiliated Wenling Hospital of Wenzhou Medical University (The First People's Hospital of Wenling), Wenling, Zhejiang Province, 317500, China; c Oujiang Laboratory (Zhejiang Lab for Regenerative Medicine, Vision, and Brain Health), National Key Laboratory of Macromolecular Drugs and Large-scale Preparation, School of Pharmaceutical Sciences, Wenzhou Medical University, Wenzhou, Zhejiang, 325035, China; d School of Rehabilitation Medicine, Wenzhou Medical University, Wenzhou, Zhejiang, 325035, China; e Department of Physical Medicine and Rehabilitation, The Second Affiliated Hospital and Yuying Children's Hospital of Wenzhou Medical University, Wenzhou, Zhejiang Province, 325000, China; f National Key Laboratory of Macromolecular Drug Development and Manufacturing, School of Pharmaceutical Science, Wenzhou Medical University, Wenzhou, 325035, China

**Keywords:** Exercise, Exosomes, Hydrogel microneedles, miR-151-3p, ROMO1

## Abstract

Exercise (Exe) training is a cornerstone of multimodal rehabilitation of patients with spinal cord injury (SCI), yet the precise mechanisms through which it exerts its therapeutic benefits remain unclear. Exosomes (Exos) are key mediators of intercellular communication and promising vehicles for targeted therapy. This study aimed to investigate the function and underlying mechanism of exercise-derived exosomes (Exe-Exos) in SCI recovery. Circulating Exos were isolated from rats subjected to a 4-week treadmill Exe regimen and from sedentary controls. A gelatin methacrylate (GelMA) hydrogel microneedles (Hyd MNs) system was developed for the targeted, sustained delivery of these Exos directly to the injury epicenter at the T10 spinal segment in a rat SCI model. Using integrated in vitro and in vivo approaches, we showed that Exe-Exos significantly promoted motor function recovery, attenuated tissue damage, reduced apoptosis, and alleviated both inflammation and oxidative stress (Oxs) after SCI. Small RNA sequencing revealed that miR-151-3p is a key functional cargo that is enriched in Exe-Exos. Gain- and loss-of-function studies revealed that exosomal miR-151-3p exerts its protective effects by directly targeting the mitochondrial membrane protein ROMO1. This targeting led to the coordinated inhibition of the pro-apoptotic JNK/Caspase pathway, suppression of the NF-κB-mediated inflammatory cascade, and activation of the Nrf2/HO-1 antioxidant axis. Collectively, our findings establish Exe-Exos, specifically exosomal miR-151-3p, as an exercise-responsive circulating signaling axis that orchestrates multifaceted protection against secondary injury after SCI, offering an innovative, mechanism-based strategy for neuroregenerative therapy.

## Highlights

• Exercise-derived exosomes (Exe-Exos) from treadmill-trained rats promote functional recovery after spinal cord injury (SCI).
• A novel hydrogel microneedles (Hyd MNs) system enables targeted, sustained delivery of Exe-Exos to the injured spinal cord.
• Exosomal miR-151-3p is identified as a key exercise-responsive miRNA mediating the therapeutic effects of Exe-Exos.
• miR-151-3p exerts anti-inflammatory, anti-apoptotic, and anti-oxidative effects by directly targeting the mitochondrial gene ROMO1.
• This study proposes an “exercise–exosome–miRNA” axis as a promising cell-free therapeutic strategy for SCI.

## Graphical abstract

Proposed mechanism by which Exe-Exos promote functional recovery after SCI. Exosomal miR-151-3p enters target cells and binds to the mitochondrial membrane gene ROMO1, leading to reduced ROS levels. This inhibits cell apoptosis through the JNK/Caspase pathway and suppresses inflammation by decreasing NF-κB-mediated transcription of pro-inflammatory factors. *Sed* sedentary*; Exe* exercise*; ROMO1* reactive oxygen species modulator 1*; ROS* reactive oxygen species*; IKK* inhibitor of kappa B kinase*,JNK* C-jun N-terminal kinase.

**Figure:**
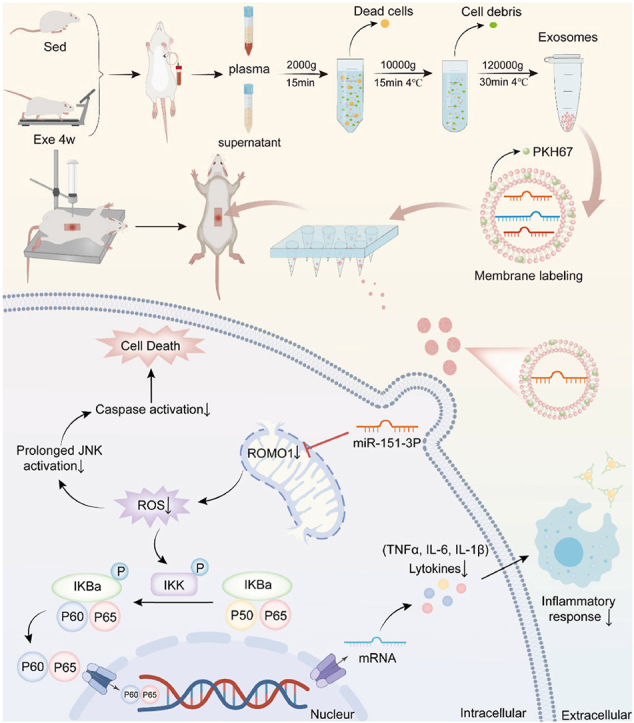

Spinal cord injury (SCI) is a devastating neurological condition with a high disability rate, imposing a severe burden on healthcare systems and society. Its incidence is increasing annually [1,2]. The pathological process of SCI comprises primary and secondary injury phases, which together lead to motor, sensory, and autonomic dysfunction [3]. The cascade of biochemical reactions triggered by primary injury progressively worsens the damage. Among these injuries, secondary injury—driven by free radical bursts, the inflammatory response, apoptosis, mitochondrial dysfunction, and axonal degeneration—plays a critical role in functional deterioration [4,5]. Currently, clinical treatments for SCI are limited and largely rely on exogenous drugs such as methylprednisolone, which are accompanied by severe side effects and poor efficacy [6,7]. Surgical intervention can only relieve spinal cord compression but cannot repair necrotic neural tissue [8,9]. Consequently, novel therapeutic strategies that can effectively halt the vicious cycle of secondary damage and promote neural repair are urgently needed.

In recent years, exosomes (Exos) research has expanded explosively from basic biology to cutting-edge applications in clinical diagnosis, therapy, drug delivery, and disease mechanism elucidation. Exos are nanoscale vesicles (30–150 nm) that are actively secreted by cells and carry proteins, nucleic acids (miRNAs, lncRNAs, and mRNAs), lipids, and metabolites from their parent cells [10,11]. They circulate in the blood, with much higher yields in plasma or serum than those secreted by a single cell type [12,13]. Exos mediate intercellular molecular transfer and communication, regulating various physiological and pathological processes [14]. Age, chronic diseases, environmental factors, and genetic disorders can disrupt this intercellular communication, impairing healing [15,16]. Recent studies have increasingly focused on Exos-mediated intercellular crosstalk [17,18]. Importantly, exercise (Exe) promotes the rapid release of Exos into the circulation [19,20]. Compared with other delivery systems, Exos are attractive drug vehicles because of their inherent selectivity, biocompatibility, stability, and long-range intercellular communication capacity [[21], [22], [23]]. A 2015 report highlighted plasma-derived therapies as a compelling approach for infectious and immune-mediated diseases [13]. Exos are rich in microRNAs (miRNAs), which are RNAs that regulate gene expression by binding to complementary sequences, thereby modulating transcriptional, translational, and epigenetic processes [[24], [25], [26], [27]]. Thus, miRNAs hold broad promise in basic research, disease diagnosis, and treatment.

Exe is a cornerstone for maintaining health, preventing disease, and promoting rehabilitation. Its mechanisms include neuromuscular adaptation, metabolic regulation, inflammation control, and gene expression modulation [28,29]. Exe is the most common and effective rehabilitation measure in clinical practice [30] and can exert protective effects through multiple targets to minimize functional damage after SCI [31,32]. Exe has been shown to upregulate endogenous neurotrophic factors and promote neurovascular and synaptic regeneration [[33], [34], [35]]. Moreover, circulating Exos levels increase immediately after Exe, and these Exos may participate in Exe-mediated adaptation processes [19,20,36]. Exe also stimulates the release of bioactive factors, including peptides and nucleic acids from skeletal muscle (myokines), adipose tissue (adipokines), and other tissues into the circulation [37]. These factors have been termed “exerkines” in the literature, and a subset may function as adipokines, contributing significantly to the systemic health benefits of Exe [[38], [39], [40]]. Our research focuses on the mechanisms by which Exe ameliorates SCI. However, given the complexity of Exe systems and the breadth of their targets, the precise mechanism remains challenging to elucidate. In this follow-up study, we extracted serum Exos from exercised rats for in-depth analysis.

A traditional tail vein injection of Exos is limited by poor targeting and a failure to achieve therapeutic concentrations at the injury site. In situ injection into spinal cord tissue leads to an uneven distribution, a lack of sustained effects, and potential damage to adjacent healthy tissue. Methacrylated gelatin (GelMA) can continuously deliver Exos to the SCI site, providing a biocompatible microenvironment for long-term treatment [[41], [42], [43]]. Our team has previously developed Exos delivery systems using porous microneedle patches for SCI intervention, and the results were published in *Nature Nanotechnology* [44] and *Nature Communications* [45]. In this study, we employed a new hydrogel microneedles (Hyd MNs) material to deliver Exe-Exos.

Building on the concepts of Exe preconditioning and Exe-induced humoral signaling [[46], [47], [48]], we hypothesized that Exe-Exos isolated from the circulation would confer superior neuroprotective effects compared with sedentary-derived Exos (Sed-Exos) when delivered via the Hyd MNs system. We further postulated that these benefits are mediated by specific miRNA cargos within Exe-Exos. Accordingly, this study aimed to: (1) compare the therapeutic efficacy of Hyd MNs-delivered Exe-Exos with that of Sed-Exos in a rat SCI model and assess the functional, electrophysiological, and histopathological outcomes; (2) identify differentially expressed miRNAs in Exe-Exos through small RNA sequencing; (3) validate the key functional miRNA, miR-151-3p, using gain- and loss-of-function approaches in vitro and in vivo; and (4) elucidate its downstream molecular target and the integrated mechanisms underlying its antiapoptotic, anti-inflammatory, and antioxidant effects.

Our integrated approach—from targeted delivery and phenotypic rescue to mechanistic dissection—provides a comprehensive evaluation of Exe-Exos as a novel therapeutic agent. We identified exosomal miR-151-3p as a central effector that targets the mitochondrial protein ROMO1, thereby coordinately modulating the JNK/Caspase, NF-κB, and Nrf2/HO-1 pathways to mitigate secondary injury. This work not only reveals a novel Exe-responsive exosomal signaling mechanism that mediates the benefits of Exe but also proposes a convergent therapeutic strategy combining a bioactive Exos cargo with an advanced delivery system for SCI repair.

## 1 Methods

### 1.1 Reagents and antibodies

Servicebio (Wuhan, China) provided the BCA protein assay (G2026), CD9 (GB155697), CD63 (GB115712), CD81 (GB111073), Calnexin (GB151369), GM130 (GB150094) and tumor susceptibility gene 101 (TSG101) (GB11618). Anti-phospho-JNK (AF3318), anti-JNK (AF6318), anti-C-Caspase9 (AF5240), anti-Caspase3 (AF6311), anti-IKK (AF6014), anti-p-IKK (AF3013), anti-p65 (AF2006), anti-IL-6 (DF6087), and anti-Caspase9 (AF6348) antibodies were procured from Affinity (OH, USA). In addition, Zenbio (Suzhou, China) supplied antibodies against β-actin (R380624) and IL-1β (511369). Antibodies against C-Caspase3 (9661S) and Nrf2 (12721S) were sourced from Cell Signaling Technology (MA, USA). HO-1 (10701) and TNF-α (60291) antibodies were obtained from Proteintech (IL, USA). The ROS Assay (S0033S) and Annexin V-FITC Apoptosis Detection (C1062L) kits were obtained from Betotime (Shanghai, China). The MDA Content Assay Kit (BC0025) was supplied by Solarbio (Beijing, China). CellTracker™ CM-DiI (C7001) was obtained from Invitrogen (CA, USA).

### 1.2 Exercise therapy

The rats were subjected to a treadmill Exe program (0° slope, 8 m/min, 30 min/day, 5 d/week) for 28 d using a ZH-PT Treadmill (Huaibei Zhenghua Bio Equipment Co., Ltd., Anhui, China) that was developed based on the results of our previous study. The treadmill Exe protocol utilized in this study was a standardized regimen previously established and validated by our group for inducing reproducible physiological adaptations in rodent models of neurological conditions [49].

### 1.3 Exosome extraction

Rats were rapidly anesthetized with isoflurane, and whole blood was collected into tubes. After standing for 15 min, the supernatant was transferred to a 50 ml tube and centrifuged (2000 × g, 15 min). The resulting supernatant was then centrifuged again (10,000 × g, 15 min, 4 °C) and filtered through a 0.22 μm PBS-pre-soaked filter. The filtrate was ultracentrifuged (120,000 × g, 30 min, 4 °C), the pellet resuspended in PBS, and ultracentrifuged again under the same conditions. The final Exos pellet was resuspended in 120 μl PBS, with a 200 μl aliquot reserved as a negative control.

### 1.4 Exosome characterization and labeling

Isolated Exos were characterized by nanoparticle tracking analysis (NTA) for size and concentration, TEM for morphology, and Western blot for positive (CD63, CD9, TSG101, CD81) and negative (Calnexin, GM130) markers. For cellular uptake studies, Exos were labeled with PKH67 (Fig. 2A) as per the manufacturer's protocol. Briefly, PKH67 was diluted in Diluent C, mixed with the Exos suspension, incubated for 10 min in the dark, and the reaction stopped with PBS. Labeled Exos were reisolated by ultracentrifugation (120,000 × g, 90 min, 4 °C) and resuspended in PBS.

### 1.5 Cell culture

PC12 (RRID: CVCL_0481) and BV2 (RRID: CVCL_0182) cell lines (ATCC) were cultured in DMEM supplemented with 10% fetal bovine serum and 1% penicillin/streptomycin at 37 °C with 5% CO_2_.

To induce inflammation, adherent cells were treated with 1 μg/ml LPS, followed by Exos addition and further incubation for 24 h.

For the Oxygen-Glucose Deprivation/Reperfusion (OGD/R) model, cells were cultured in glucose-free DMEM and placed in a tri-gas incubator (1% O_2_, 5% CO_2_, 94% N_2_) for 24 h, then returned to normal complete medium under normoxic conditions (37 °C, 5% CO_2_, 95% air) for another 24 h. Model establishment was confirmed by reduced cell viability and increased LDH release compared with normoxic controls.

### 1.6 Cell flow detection

After the experimental treatments, the cells were detached with trypsin (without EDTA) and centrifuged. The supernatant was carefully removed, and the cell pellets were resuspended. For the apoptosis analysis, the cells were stained with Annexin V-FITC in dark at 37 °C for 15 min, after which flow cytometry was used to assess cell viability.

### 1.7 CM-Dil staining

Following the experimental treatments, the cells were rinsed three times with PBS. For membrane labeling, the cells were incubated with a CM-Dil working solution at 37 °C in the dark for 5 min, followed by immediate transfer to 4 °C for another 20 min of incubation. After washes with PBS to remove the excess dye, the cells were immediately analyzed using a laser-scanning confocal microscope.

### 1.8 Dihydroethidium (DHE) staining

After treatment, the culture medium was aspirated, and the cells were rinsed three times with PBS. DCFH-DA working solution was then added to each well, followed by incubation at 37 °C in dark for half 1 h. After staining, the cells were rinsed with PBS to eliminate the excess probe, and fluorescence was immediately observed with a fluorescence microscope.

### 1.9 Fabrication of the hydrogel microneedles patch for exosome delivery

Gelatin methacrylate (GelMA) was synthesized by reacting gelatin with methacrylic anhydride (MAA) in bicarbonate buffer at 45 °C. For sterile preparation, GelMA powder was dissolved in sterile ddH₂O and filter-sterilized. Exos were extracted under sterile conditions, resuspended in sterile PBS, filtered (0.22 μm), and verified sterile by LB agar plating. The PDMS mold was sterilized with 75% ethanol (30 min), rinsed with sterile PBS, and UV-treated. Inside a biosafety cabinet, sterile GelMA precursor (10% w/v) containing photoinitiator was mixed with sterile Exos, cast into the mold, degassed, and UV-crosslinked (405 nm, 60 s). A 20% PVA backing layer was added and dried at 30–35 °C. All animal surgeries and microneedle implantations were performed in a sterile operating room with disinfected skin and sterile instruments. Microneedles were implanted into the injured T10 spinal cord using sterile forceps, and the wound was sutured and disinfected daily.

### 1.10 In vitro release kinetics of exosomes from hydrogel microneedles

PKH67-labeled Exe-Exos were loaded into Hyd MNs as described above. Each patch was immersed in 1 ml PBS (pH 7.4) containing 0.5% BSA and incubated at 37 °C with gentle shaking (60 rpm). At designated time points (1, 3, 6, 12, 24, 48, 72, 96, 120, 144, and 168 h), the entire release medium was collected and replaced with fresh buffer. Fluorescence intensity of the collected medium was measured and converted to Exos quantity using a standard curve. Cumulative release percentage was calculated based on the initial loading. Three independent patches were tested per time point.

### 1.11 Mechanical characterization and penetration performance of hydrogel microneedles

Mechanical strength was evaluated using a universal testing machine (INSTRON, MA, USA) by compressing the microneedle array with a cylindrical probe at 0.5 mm/s to a displacement of 3.5 mm; the failure force per needle was determined from the force–displacement curves. For penetration depth, Hyd MNs were pressed into freshly excised rat spinal cord (T10) for 30 s, after which the tissue was fixed, sectioned, and stained with H&E.

### 1.12 Animals and experimental groups

A total of 240 adult male SD rats (200–250 g) were randomly assigned into eight groups (n = 30 per group): Sham, SCI, SCI + Hyd MNs, SCI + Hyd MNs@Sed-Exos, SCI + Hyd MNs@Exe-Exos, SCI + Exos + NC-antagomir, and SCI + Exos + miR-151-3p-antagomir. All experimental procedures were approved by the Animal Research Committee of Wenzhou Medical University (reference No. xmsq2023-0084) and complied with the NIH Guide for the Care and Use of Laboratory Animals.

### 1.13 Spinal cord injury model and hydrogel microneedles implantation

A rat SCI model was induced at the T10 vertebral level using a New York University Impactor (10 g weight dropped from a 20 mm height) under pentobarbital sodium anesthesia (30 mg/kg, i.p.). Immediately after injury, the dura was gently exposed, and the Exos-loaded or empty Hyd MNs were implanted directly onto the dorsal surface of the injured spinal cord (Fig. 3G–H). The muscle and skin were sutured in layers. Postoperative care included manual bladder expression twice daily.

### 1.14 Functional behavioral evaluation

Hindlimb motor function recovery was assessed at 1, 3, 7, 14, and 28 days post injury (DPI) using the Basso–Beattie–Bresnahan (BBB) scale [45,50]. For the electrophysiological assessment, the spinal cord was stimulated electrically (10 mA, 0.1 ms pulse duration, 1 Hz) once every 15 s, and the amplitude of evoked potentials was recorded. All behavioral and electrophysiological data were collected by two independent observers who were blinded to the treatment groups.

### 1.15 Application of microRNA inhibitors and microRNA antagomir injection

GenePharma (Shanghai, China) synthesized the miR-151-3p inhibitor (5′-CCUCAAGGAGCCUCAGUCUAG-3′) and the corresponding antagomir (same sequence). For in vitro experiments, 40 μg of Exos were mixed with 2 μM miR-151-3p inhibitor. For in vivo experiments, 1 nmol of miR-151-3p antagomir or NC antagomir was injected intrathecally once daily for 5 consecutive days (Fig. 8A). All experiments were performed by an investigator who was blinded to group assignments to minimize bias.

### 1.16 Tissue preparation

Rats were euthanized at predetermined time points post-SCI. Spinal cord tissues were quickly dissected, fixed in fixative solution for 24 h at 4 °C, then cryoprotected in 20% and 30% sucrose in PBS (overnight each at 4 °C), and sectioned into 15-μm thick slices using a cryostat for subsequent analyses.

### 1.17 Masson's trichrome staining

Fixed sections were rinsed for 15 min, stained overnight in Masson's A solution, then washed. Sections were placed in Masson's B/C mixture for 1 min, rinsed, briefly differentiated, and washed again. They were then incubated in Masson's D for 6 min, rinsed, incubated in Masson's E for 1 min, and dried. Masson's F was applied for 2–30 s, followed by washing, differentiation with 1% acetic acid, and two dehydrations in anhydrous ethanol. A third dehydration (5 min), clearing in xylene (5 min), and mounting with neutral gum were performed. Stained sections were examined microscopically.

### 1.18 Hematoxylin and Eosin (H&E) staining and glycine silver staining

Prepared slices (n = 5) were air-dried for 3 min, stained with H&E, and imaged using an Olympus BH-2 microscope (Olympus Optics, London, UK).

Glycine silver staining was performed as previously described [50]. Briefly, deparaffinized and rehydrated sections were washed in distilled water for 20 min, incubated in silver glycine solution C for 5 min, then treated with solution B for 3–5 min. Sections were transferred to preheated (45 °C) solution AI for a few seconds, replaced with solution AII, dehydrated through graded ethanol, cleared in xylene, mounted with neutral balsam resin, and examined microscopically.

### 1.19 TUNEL assay

Slides (n = 5 per group) were permeabilized for 10 min at room temperature, rinsed three times (15 min each), and apoptotic cells were detected using an In Situ Cell Death Detection Kit (Roche). Fluorescence images were acquired with a BX51 microscope (Olympus), and apoptosis-positive cells were quantified using Image-Pro Plus 6.0 software.

### 1.20 Small RNA sequencing

Small RNA libraries were prepared and sequenced by OE Biotech Co., Ltd. (Shanghai, China). Total RNA was extracted using TRIzol, quantified with a Nanodrop 2000, and assessed for integrity using an Agilent 2100 Bioanalyzer. Libraries were constructed from 1 μg total RNA using the NEBNext Small RNA Library Prep Set (NEB) and size-selected (140–160 bp). Sequencing was performed on an Illumina NovaSeq 6000 platform (150 bp paired-end). Raw reads were trimmed with cutadapt (v1.14), and reads of 18–35 nt were retained. Filtered reads were aligned to the rat reference genome (mRatBN7.2) using Bowtie2 (v1.1.1), and miRNA counts were quantified with featureCounts based on miRBase (v22). Normalization was performed using TPM. Differential expression analysis was conducted with DESeq2 (v1.22.2), and p-values were adjusted by the Benjamini-Hochberg method. miRNAs with an adjusted p value (q value) < 0.05 and |log_2_FC| > 1 were considered differentially expressed.

### 1.21 Real-time fluorescence quantitative PCR

Total exosomal RNA was extracted using a TRIzol kit (Pufei, Shanghai, China) according to the manufacturer's instructions. After centrifugation, the RNA precipitate was dissolved in RNase-free water, quantified using a NanoDrop 2000/2000C (Thermo, MA, USA), and reverse-transcribed into cDNA using the M-MLV kit (Promega, WI, USA). Relative gene expression was calculated by the 2^−ΔΔCt^ method. The miRNA stem-loop primers (Shanghai Jierui Bioengineering) are listed in Table 1. Differential expression of key miRNAs (e.g., miR-151-3p) was validated by RT-qPCR using RNA from an independent batch of Exos samples (biological replicates), confirming the reproducibility of the sequencing results.

**Table 1:** miRNA sequence information.

Gene	Forward primer	Reverse primer
rno-miR151-3p	CGCGCTAGACTGAGGCTCC	AGTGCAGGGTCCGAGGTATT
rno-miR-486	CGCGTCCTGTACTGAGCTGC	AGTGCAGGGTCCGAGGTATT
rno-miR-382-5p	GCGGAAGTTGTTCGTGGTG	AGTGCAGGGTCCGAGGTATT
rno-miR-122-5p	CGCGTGGAGTGTGACAATGG	AGTGCAGGGTCCGAGGTATT
rno-miR-423-5p	TGAGGGGCAGAGAGCGAG	AGTGCAGGGTCCGAGGTATT
rno-let-7d-3p	GCGCTATACGACCTGCTGC	AGTGCAGGGTCCGAGGTATT
rno-miR-28-3p	CGCGCACTAGATTGTGAGCT	AGTGCAGGGTCCGAGGTATT
rno-miR-375-3p	GCGTTTGTTCGTTCGGCTC	AGTGCAGGGTCCGAGGTATT
rno-miR-187-3p	CGCGTCGTGTCTTGTGTTGC	AGTGCAGGGTCCGAGGTATT
rno-miR-206-3p	GCGCGTGGAATGTAAGGAAGT	AGTGCAGGGTCCGAGGTATT
rno-miR-125a-3p	CGCGACAGGTGAGGTTCTTG	AGTGCAGGGTCCGAGGTATT
Rat-U6	CTCGCTTCGGCAGCACA	AACGCTTCACGAATTTGCGT

### 1.22 WB analysis

Protein concentrations were determined using a BCA assay. Proteins were separated by SDS-PAGE, transferred onto PVDF membranes (Bio-Rad), blocked in 5% milk for 90 min, and incubated overnight at 4 °C with primary antibodies against CD63 (1:1000), CD9 (1:1000), TSG101 (1:1000), CD81 (1:1000), p-JNK (1:1000), JNK (1:1000), C-Caspase9 (1:1000), Caspase9 (1:500), C-Caspase3 (1:1000), Caspase3 (1:500), Nrf2 (1:1000), HO-1 (1:1000), IKK (1:1000), p-p65 (1:1000), IL-6 (1:1000), TNF-α (1:1000), IL-1β (1:1000), ROMO1 (1:1000), and β-actin (1:1000). Membranes were then incubated with secondary antibodies for 2 h. Immunoreactive bands were visualized using a ChemiDoc™ XRS + Imaging System (Bio-Rad).

### 1.23 Immunofluorescence staining

Sections were incubated overnight at 4 °C with primary antibodies against Nrf2 (1:200), HO-1 (1:200), TNF-α (1:500), and p-p65 (1:500), followed by incubation with corresponding secondary antibodies for 50 min at room temperature. Nuclei were counterstained with DAPI. For each group, five random fields per slide were captured from three replicate slides, and fluorescence intensity was analyzed using ImageJ software (NIH, Bethesda, MD, USA).

### 1.24 Plasmid construction and transfection

The ROMO1 overexpression plasmid contained the SV40-firefly_Luciferase-MCS element, and the rno-miR-151 plasmid contained the CMV enhancer-MCS-polyA-EF1A-zsGreen-sv40-puromycin element. 293T cells were thawed, centrifuged (1300 rpm, 3 min), and resuspended in complete medium. Cells were seeded into 6 cm dishes and cultured at 37 °C with 5% CO_2_ until reaching ∼90% confluence, then passaged. Transfection was performed using Lipofectamine 2000 (Invitrogen) according to the manufacturer's protocol. After 24–48 h, >80% of cells expressed the fluorescent marker; 500 μl of fresh medium was added, and cells were cultured to 100% confluence before collection for RNA or protein analysis. RT-PCR was performed as described above.

### 1.25 Luciferase reporter assay

Luciferase activity was measured using the Dual-Luciferase® Reporter Assay System (GenePharma, Shanghai, China). 293T cells were transfected with the reporter vector, and lysed 48 h post-transfection. Firefly luciferase activity was measured after adding 100 μl of substrate to 20 μl of lysate, followed by Renilla luciferase activity after adding 100 μl of Renilla substrate and incubating for 10 min. Relative luciferase activity was calculated as the firefly/Renilla ratio.

### 1.26 Transmission Electron Microscopy (TEM) of mitochondria

Fresh spinal cord tissues were rapidly dissected into 1 mm^3^ pieces, fixed in 2.5% glutaraldehyde, post-fixed in 1% osmium tetroxide for 1 h, and stained with 1% uranyl acetate for 2 h. Samples were then dehydrated through an acetone gradient and embedded for coronal sectioning. The target region was identified by semithin sectioning and toluidine blue staining, followed by ultrathin sectioning and examination using a Hitachi TEM.

### 1.27 Statistical analysis

Data are presented as means ± SEM. Normality was assessed using the Shapiro–Wilk test (*p* > 0.05 considered normal) [51], and homogeneity of variances by Levene's test (*p* > 0.05 considered equal). Two-group comparisons were performed using unpaired Student's t-test. For multiple groups, two-way ANOVA followed by Tukey's post hoc test was used. Behavioral data (repeated measures) were analyzed by two-way repeated-measures ANOVA with group as between-subjects factor and time as within-subjects factor, followed by Tukey's test. Sphericity was checked with Mauchly's test; if violated, the Greenhouse–Geisser correction was applied. All datasets met the homogeneity of variances assumption. Statistical analyses were performed using SPSS 16.0, with significance set at *p* < 0.05. Effect sizes (Cohen's ∗d∗) are reported for key comparisons. Significance levels: n.s. >0.05, ∗*p* < 0.05; ∗∗*p* < 0.01; ∗∗∗*p* < 0.001.

### 1.28 Experimental replicates

All the quantitative results are derived from independent biological replicates. The reported sample size (e.g., n = 5) in the text and figure legends refers to the number of individual animals (in vivo) or independent cell culture preparations (in vitro), not to technical replicates. This design ensures biological relevance and statistical robustness.

## 2 Results

### 2.1 Extraction and characterization of Exe-Exos

**Fig. 1:**
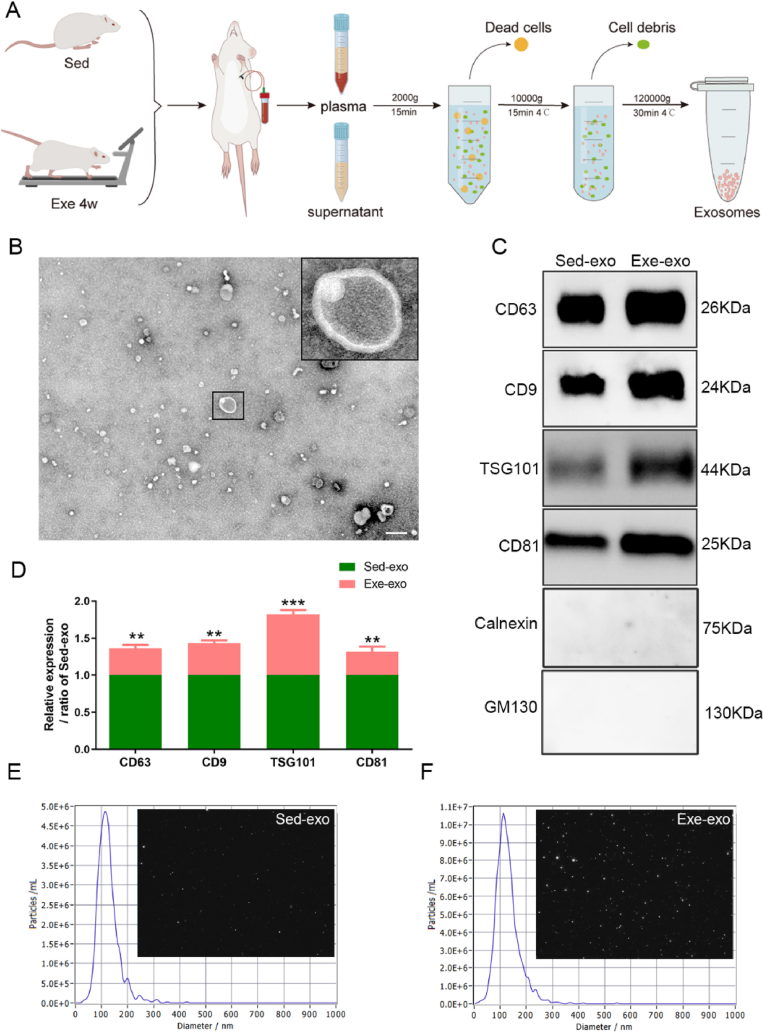
**Extraction and characterization of Exe-Exos. (A)** Flow chart of Exos extraction. **(B)** TEM images of Exos ultrastructure (enlarged view inset). Scale bar = 100 nm. **(C)** WB analysis and **(D)** quantification of CD63, CD9, TSG101, and CD81 protein expression in Sed-Exos and Exe-Exos groups (n = 5 independent biological replicates). **(E**–**F)** NTA showing size distribution of serum-purified Exos from Sed and Exe rats. Particle concentration was measured from n = 5 independent serum samples (biological replicates). *Sed* sedentary, *Exe* exercise, *TEM* Transmission electron microscopy, *NTA* Nanoparticle tracking analysis.

To investigate the effect of Exe on circulating Exos, we isolated Exos from the serum of Sed and 4-week treadmill-Exe rats by differential ultracentrifugation (Fig. 1A). TEM revealed typical cup-shaped vesicles of 100–130 nm in both groups (Fig. 1B). Western blot confirmed the presence of exosomal markers (CD63, CD9, TSG101, and CD81) and absence of endoplasmic reticulum (Calnexin) and Golgi (GM130) markers (Fig. 1C and D). Protein levels of these markers were significantly higher in Exe-Exos than in Sed-Exos (CD63, *p* < 0.01; CD9, *p* < 0.01; TSG101, *p* < 0.001; CD81, *p* < 0.01). NTA further showed a higher particle concentration for Exe-Exos (1.05 × 10^7^/ml) than for Sed-Exos (4.8 × 10^6^/ml), with a similar modal size of ∼110 nm in both groups (Fig. 1E and F). These results indicate that chronic Exe increases the basal level of circulating Exos without altering their fundamental physical characteristics.

**Fig. 2:**
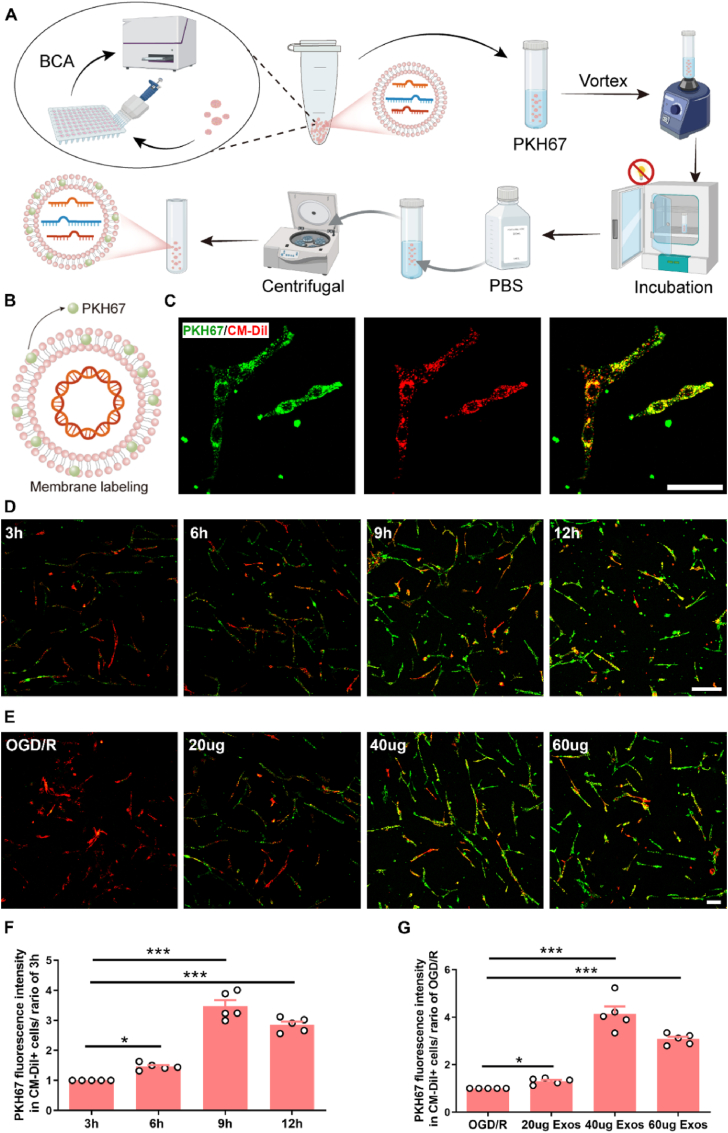
**Optimized cellular uptake of Exe-Exos. (A)** Schematic of PKH67 membrane labeling of Exos. **(B**–**E)** Double immunofluorescence staining of PKH67 (green) and CM-DiI (red) showing Exos internalization. Scale bars: 50, 200, and 100 μm, respectively. **(F**–**G)** Quantification of PKH67 fluorescence intensity in CM-DiI^+^ cells. n = 5 independent biological replicates (cell culture preparations). *BCA* bicinchoninic acid, *PBS* phosphate buffered saline, *h* hour, *Exos* exosomes.

**Fig. 3:**
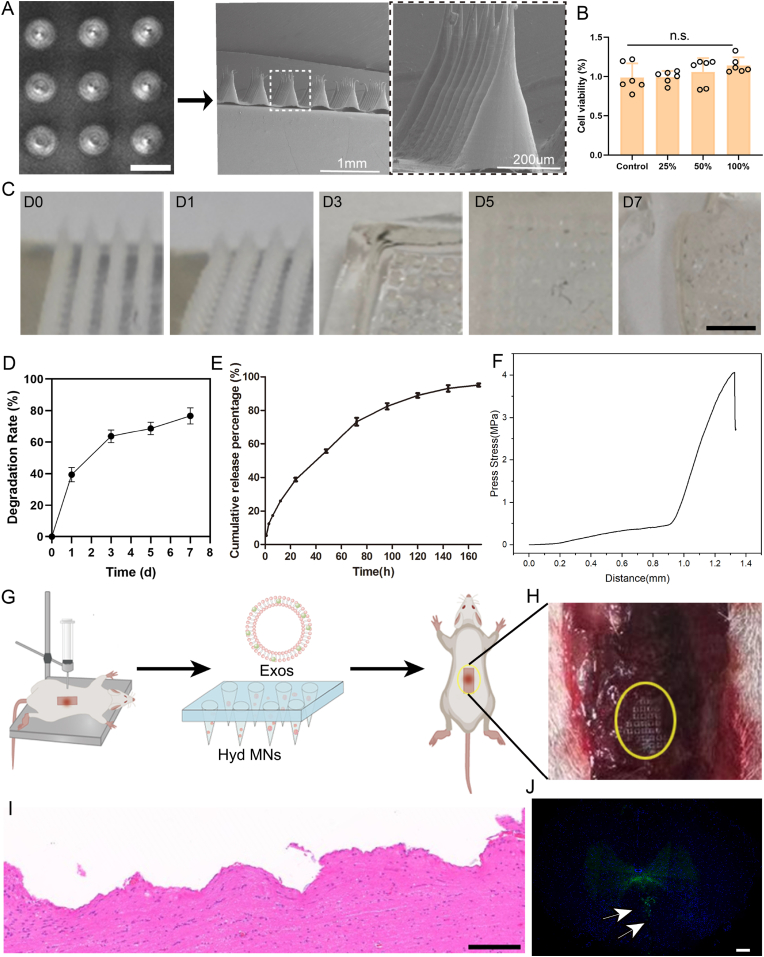
**Fabrication, in vivo implantation, and biocompatibility assessment of the Exos-loaded Hyd MNs. (A)** Schematic illustration of Exos encapsulated within the GelMA-based Hyd MNs array (scale bar = 0.4 mm). Macroscopic and magnified views of the fabricated Hyd MNs (scale bars: 1 mm and 200 μm). **(B)** Quantitative assessment of cell viability. n = 6 independent biological replicates (cell culture preparations). **(C**–**D)** Subsequent MNs analyses (n = 3 independent Hyd MNs, scale bar = 2 mm). **(E)** Release kinetics of Exos from the Hyd MNs patch, shown as cumulative fluorescence intensity over time. n = 3 independent patches per time point. **(F)** Pressure-displacement curve of the Hyd MNs, with compression displacement (mm) on the x-axis and applied pressure (MPa) on the y-axis. **(G**–**H)** Surgical procedure for SCI induction and in situ implantation of the Exos-loaded Hyd MNs. **(I**–**J)** H&E-stained spinal cord cross-section and PKH67 fluorescence tracking of Exos in transverse sections at the injury epicenter (scale bar = 200 μm). These data are manuscript-specific, obtained from the Hyd MNs fabricated in this study. *Exos* exosomes, *Hyd MNs* hydrogel microneedles, *SCI* spinal cord injury.

### 2.2 Optimized cellular uptake of Exe-Exos and targeted delivery via hydrogel microneedles

To guide therapeutic application, we first determined optimal conditions for Exos internalization in vitro. PKH67-labeled Exos (Fig. 2A) were applied to PC12 cells subjected to OGD/R, and uptake assessed by co-localization with CM-DiI (Fig. 2B–D). Internalization peaked at 9 h (6 h *vs*. 3 h: *p* < 0.05; 9 h *vs*. 3 h: *p* < 0.001; 12 h *vs*. 3 h: *p* < 0.001; Fig. 2E) and was most efficient at 40 μg (20 μg: *p* < 0.05; 40 μg: *p* < 0.001; 60 μg: *p* < 0.001; Fig. 2F). For targeted in vivo delivery, we fabricated GelMA Hyd MNs with sharp conical tips (∼1 mm height, 200 μm tip diameter; Fig. 3A). The patch exhibited excellent cytocompatibility, as PC12 cell viability remained unchanged across all tested extract concentrations (25–100%; Fig. 3B). In vitro degradation reached ∼85% by day 7 (Fig. 3C and D), and cumulative Exos release showed an initial burst of ∼40% within 20 h, reaching ∼95% by 168 h (Fig. 3E), confirming sustained local delivery. Mechanical testing (Instron) generated a pressure–displacement curve: pressure rose gradually from 0 to 0.40 MPa up to 0.9 mm displacement, then sharply to 3.9 MPa at 1.3 mm, where a distinct inflection indicated fracture (Fig. 3F). The average fracture force per needle (∼0.85 N) far exceeded the force needed to penetrate rat spinal dura mater (∼0.15 N), demonstrating sufficient mechanical strength for in vivo use. Following standardized T10 contusion injury (Fig. 3G), Exo-loaded Hyd MNs were implanted onto the dura mater (Fig. 3H). H&E staining showed that needles penetrated the dura mater to a depth of 300–400 μm, sufficient to reach the injury center (Fig. 3I). PKH67 fluorescence tracking confirmed successful Exos delivery into the parenchyma, including areas around the central canal (Fig. 3J).

### 2.3 Exe-Exos promote functional recovery and neural reconnection after SCI

**Fig. 4:**
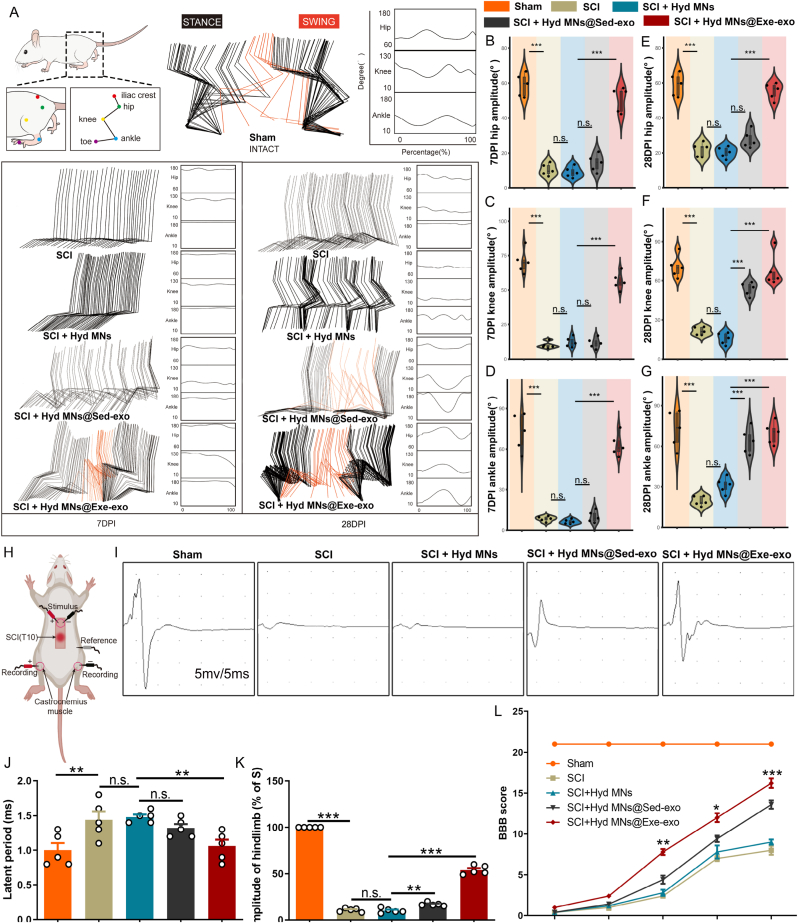
**Exe-Exos promote functional recovery and neural reconnection after SCI. (A)** Schematic diagrams of rat hip, knee, and ankle joints with color-coded stick figures representing hindlimb movements in the Sham, SCI, SCI + Hyd MNs, SCI + Hyd MNs + Sed-Exos, and SCI + Hyd MNs + Exe-Exos groups. **(B**–**G)** Amplitude analysis of hip (**B, E**), knee (**C, F**), and ankle (**D, G**) angles (n = 5 independent biological replicates). **(H)** Schematic of electrophysiological detection in SCI rats. **(I)** Electrophysiological recordings at 28 DPI (horizontal and vertical divisions: 5 ms and 5 mV, respectively). **(J**–**K)** Quantitative analysis: average amplitude relative to Sham (**J**) and latency periods (**K**) (n = 5 independent biological replicates). **(L)** BBB scores of each group at indicated time points (n = 5 independent biological replicates). Data are presented as mean ± SEM. Statistical analysis was performed using repeated-measures two-way ANOVA with Greenhouse–Geisser correction when appropriate, followed by Tukey's post hoc test. *SE* standard error, *DPI* day post injury, *SCI* spinal cord injury, *Hyd MNs* hydrogel microneedles, *Exos* exosomes, *Sed* sedentary, *Exe* exercise.

To verify the effect of Exos on motor function, we analyzed hip, knee, and ankle joint mobility at 7 and 28 DPI (Fig. 4A–G). At both time points, the SCI + Hyd MNs@Exe-Exos group showed significantly better joint mobility than the SCI + Hyd MNs group (7 d or 28 d: hip amplitude, *p* < 0.001, Cohen's d > 2.5; knee amplitude, *p* < 0.001, Cohen's d > 2.8; ankle amplitude, *p* < 0.001, Cohen's d > 2.3), with hip and knee angles approaching normal values. The SCI + Hyd MNs@Sed-Exos group showed a slight, non-significant improvement at day 7 (*p* > 0.05), but by day 28, both knee and ankle angles were significantly improved compared with SCI + Hyd MNs (knee: *p* < 0.001; ankle: *p* < 0.01). No significant differences were observed between SCI and SCI + Hyd MNs groups at either time point, and both exhibited dragging and foot dropping.

Motor evoked potentials (MEPs) were recorded to assess neural transmission (Fig. 4H–K). Compared with Sham, SCI rats showed significantly prolonged latency and reduced amplitude (latent period: *p* < 0.01; amplitude: *p* < 0.001). The SCI + Hyd MNs group did not differ from the SCI group. In contrast, the SCI + Hyd MNs@Exe-Exos group exhibited significantly improved signal transmission, with shortened latency (*p* < 0.001, Cohen's d > 2.1) and increased amplitude (*p* < 0.01, Cohen's d > 1.8).

The BBB score was evaluated at 1, 3, 7, 14, and 28 DPI (Fig. 4L). At 7 and 14 DPI, the SCI + Hyd MNs@Exe-Exos group had significantly higher scores than the SCI + Hyd MNs group (*p* < 0.01), while other groups showed no significant differences. At 28 DPI, the SCI + Hyd MNs@Exe-Exos group displayed the most pronounced recovery (*p* < 0.001, Cohen's d > 2.0). Collectively, these results indicate that Exe-Exos promote motor function recovery through neural circuit reconstruction.

### 2.4 Exe-Exos attenuate tissue destruction, collagen deposition, nerve fiber injury, and cell apoptosis in rats after SCI

**Fig. 5:**
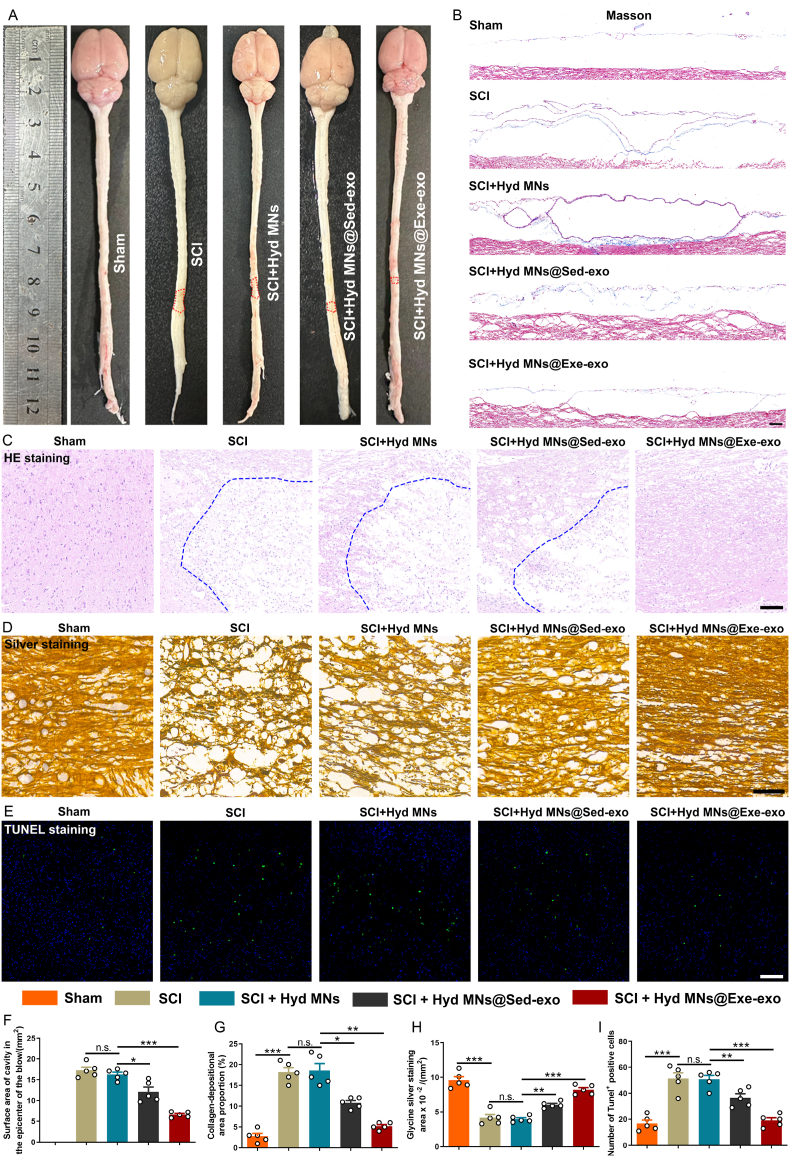
**Exe-Exos attenuate tissue destruction, collagen deposition, nerve fiber injury, and cell apoptosis in rats after SCI. (A)** Representative images of rat brain-spinal cord tissues; red dotted frames indicate the in situ injury area in the Sham, SCI, SCI + Hyd MNs, SCI + Hyd MNs + Sed-Exos, and SCI + Hyd MNs + Exe-Exos groups. **(B)** Masson staining. **(C)** H&E staining of cross-sections at 28 DPI; blue dotted lines delineate the cavity area. **(D)** Glycine silver staining of nerve fibers. **(E)** TUNEL staining showing neuronal apoptosis at the injury epicenter. **(F**–**I)** Quantification of cavity area (mm^2^, **F**), collagen deposition proportion (**G**), glycine silver-positive area (/mm^2^, **H**), and TUNEL^+^ cells (**I**); n = 5 independent biological replicates. Scale bar = 50 μm for all panels. *HE* hematoxylin–eosin, *SE* standard error, *DPI* days post injury, *SCI* spinal cord injury, *Hyd MNs* hydrogel microneedles, *Exos* exosomes, *Sed* sedentary, *Exe* exercise, *TUNEL* TdT-mediated dUTP nick end labeling.

To assess tissue damage at 28 d post-SCI, spinal cord tissues were collected, and the lesion surface was examined. Masson and H&E staining (Fig. 5A–C) showed intact structure in the Sham group, whereas SCI induced significant cavity formation and collagen deposition (*p* < 0.001). Exos treatment markedly reduced these effects, decreasing glial scar area (cavity: *p* < 0.001, Cohen's d > 2.5; collagen deposition ratio*: p* < 0.01, Cohen's d > 1.9; Fig. 5F and G). Glycine silver staining (Fig. 5D) revealed evenly distributed, regularly arranged nerve fibers in Sham rats, while SCI caused axonal swelling, distortion, breakage, and retraction (SCI *vs*. Sham: *p* < 0.001). The SCI + Hyd MNs group showed no improvement over SCI (*p* > 0.05), whereas the Hyd MNs@Sed-Exos group exhibited some fiber recovery, and the Hyd MNs@Exe-Exos group showed significant improvement (Hyd MNs@Exe-Exos *vs*. SCI + Hyd MNs, *p* < 0.001, Cohen's d > 2.7; Fig. 5H). TUNEL staining (Fig. 5E) demonstrated that the number of apoptotic cells at the injury epicenter was markedly increased in the SCI and SCI + Hyd MNs groups compared with Sham (SCI and SCI + Hyd MNs *vs*. Sham, *p* < 0.001), with no difference between SCI and SCI + Hyd MNs (*p* > 0.05). Both Exos-treated groups showed significantly fewer apoptotic cells, with a greater reduction in the SCI + Hyd MNs@Exe-Exos group (SCI + Hyd MNs@Sed-Exos *vs*. SCI, *p* < 0.01; SCI + Hyd MNs@Exe-Exos *vs*. SCI, *p* < 0.001, Cohen's d > 2.4; Fig. 5I).

### 2.5 miR-151-3p is a key functional cargo in Exe-Exos mediating anti-apoptotic and antioxidant effects

**Fig. 6:**
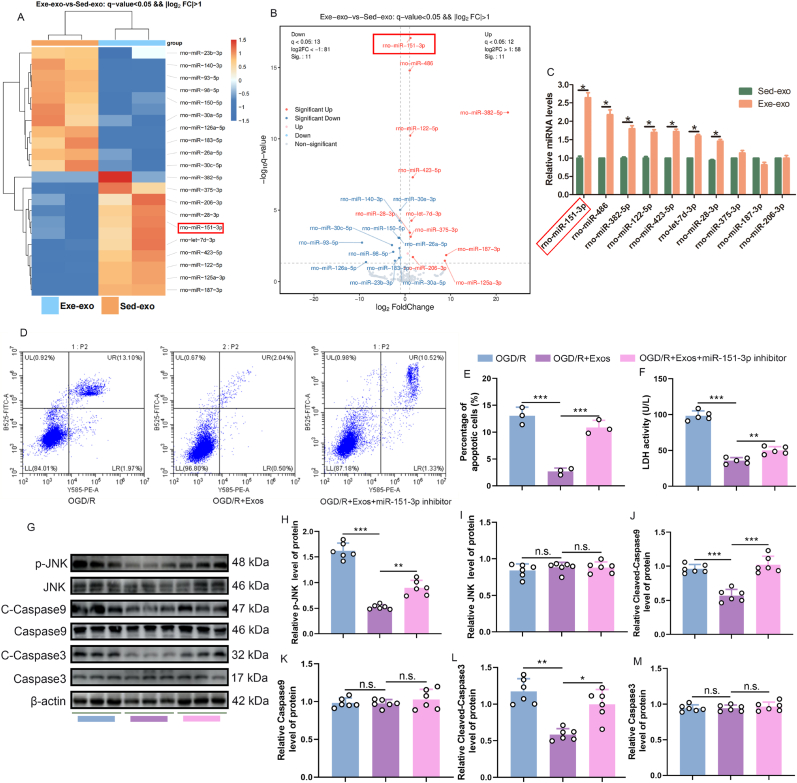
**miR-151-3p is a key functional cargo in Exe-Exos mediating anti-apoptotic and antioxidant effects. (A**–**B)** Differentially expressed miRNAs between Exe-Exos and Sed-Exos groups (q < 0.05, |log_2_FC| > 1). **(C)** RT-qPCR validation of 10 differentially expressed miRNAs. **(D)** Flow cytometry analysis of apoptosis in OGD/R, OGD/R + Exos, and OGD/R + Exos + miR-151-3p groups. **(E)** Quantification of apoptotic cell percentage (n = 3 independent cell culture experiments). **(F)** LDH activity (U/L; n = 5 independent cell culture experiments). **(G)** WB and **(H**–**M)** quantification of p-JNK, JNK, C-Caspase9, Caspase9, C-Caspase3, and Caspase3 protein expression (n = 6 independent biological replicates). *Exos* exosomes, *JNK* c-Jun N-terminal kinase.

To identify key neuroprotective microRNAs in Exe-Exos, we performed small RNA sequencing on Exe-Exos and Sed-Exos. Twenty miRNAs were significantly differentially expressed (|log_2_FC| > 1, q < 0.05; Fig. 6A and B and Table 2), of which ten were upregulated in Exe-Exos. RT-qPCR confirmed that miR-151-3p was significantly overexpressed in Exe-Exos (Exe-Exos *vs*. Sed-Exos, *p* < 0.05; Fig. 6C). Flow cytometry showed that Exe-Exos markedly reduced OGD/R-induced apoptosis, whereas the miR-151-3p inhibitor reversed this protection (both comparisons *p* < 0.001; Fig. 6D and E). Consistently, miR-151-3p significantly decreased LDH release, an effect blocked by the inhibitor (OGD/R + Exos vs. OGD/R: *p* < 0.001; OGD/R + Exos + miR-151-3p inhibitor *vs*. OGD/R + Exos, *p* < 0.01; Fig. 6F). Western blot analysis revealed that Exe-Exos downregulated p-JNK, C-Caspase9, and C-Caspase3, effects reversed by the miR-151-3p inhibitor (Fig. 6G–M). In contrast, total JNK, Caspase9, and Caspase3 levels remained unchanged (all *p* > 0.05), indicating that Exe-Exos modulate the activated forms without affecting total protein expression.

**Table 2:** miRNA expression.

miRNA_id	FoldChange	log2FoldChange	p-value	q-value	Regulation	Sequence
rno-let-7d-3p	2.6739434292774904	1.41896894367953	1.7329457535893996e-06	5.46096089801745e-05	Up	CTATACGACCTGCTGCCTTTCT
rno-miR-122-5p	2.1522935121388	1.10587483422937	1.02793356959666e-12	5.936316364420729e-11	Up	TGGAGTGTGACAATGGTGTTTG
rno-miR-151-3p	2.26791546744031	1.181366867362389	3.750656449028e-20	8.66401639725469e-18	Up	CTAGACTGAGGCTCCTTGAGG
rno-miR-187-3p	521.842199318741	9.02746980370053	0.00114244962590086	0.0138897822938473	Up	TCGTGTCTTGTGTTGCAGCCGG
rno-miR-206-3p	3.3078733384911	1.725903993448350	0.00165292451798031	0.0190912781826725	Up	TGGAATGTAAGGAAGTGTGTGG
rno-miR-28-3p	2.08750205986828	1.06177762118443	1.62309109259892e-05	0.000374934042390351	Up	CACTAGATTGTGAGCTCCTGGA
rno-miR-375-3p	2.3191713042349704	1.21360938774702	3.45162354167105e-05	0.00072484094375092	Up	TTTGTTCGTTCGGCTCGCGTGA
rno-miR-382-5p	6246370.64099003	22.5745867464402	1.86730333091614e-14	1.4378235648054296e-12	Up	GAAGTTGTTCGTGGTGGATTCG
rno-miR-423-5p	3.23674443614577	1.6945434590718	1.0581747441534698e-09	4.88876731798904e-08	Up	TGAGGGGCAGAGAGCGAGACTTTT
rno-miR-486	2.04195690314573	1.02995241750168	1.3745970835513199e-17	1.58765963150177e-15	Up	TCCTGTACTGAGCTGCCCCGAG
rno-miR-126a-5p	0.0026488664138472	−8.560409196	0.00461123553876931	0.0426078163782284	Down	CATTATTACTTTTGGTACGCG
rno-miR-140-3p	0.441648503408707	−1.179029472	1.89124186944327e-06	5.46096089801745e-05	Down	TACCACAGGGTAGAACCACGG
rno-miR-150-5p	0.377437618745349	−1.405689873	4.83037329436658e-05	0.000858320177691293	Down	TCTCCCAACCCTTGTACCAGTG
rno-miR-183-5p	0.40580971715843	−1.301124684	0.00206226590621891	0.0216537920152985	Down	TATGGCACTGGTAGAATTCACT
rno-miR-23b-3p	0.214492678485719	−2.220999691	0.00348698869899619	0.0342877431396509	Down	ATCACATTGCCAGGGATTACC
rno-miR-26a-5p	0.4270478355364961	−1.227530413	4.49166540606334e-05	0.000858320177691293	Down	TTCAAGTAATCCAGGATAGGCT
rno-miR-30a-5p	0.42928424399473103	−1.219994872	0.000315633006412755	0.00455695153008415	Down	TGTAAACATCCTCGACTGGAAG
rno-miR-30c-5p	0.13687304249379	−2.869089763	0.000179912419740202	0.00277065126399911	Down	TGTAAACATCCTACACTCTCAGC
rno-miR-93-5p	0.00144804365063188	−9.431679192	0.000113360685401675	0.0018704513091276301	Down	CAAAGTGCTGTTCGTGCAGGTAG
rno-miR-98-5p	0.16597612297760506	−2.590952382	0.000605661310175408	0.00822986839120702	Down	TGAGGTAGTAAGTTGTATTGTT

**Fig. 7:**
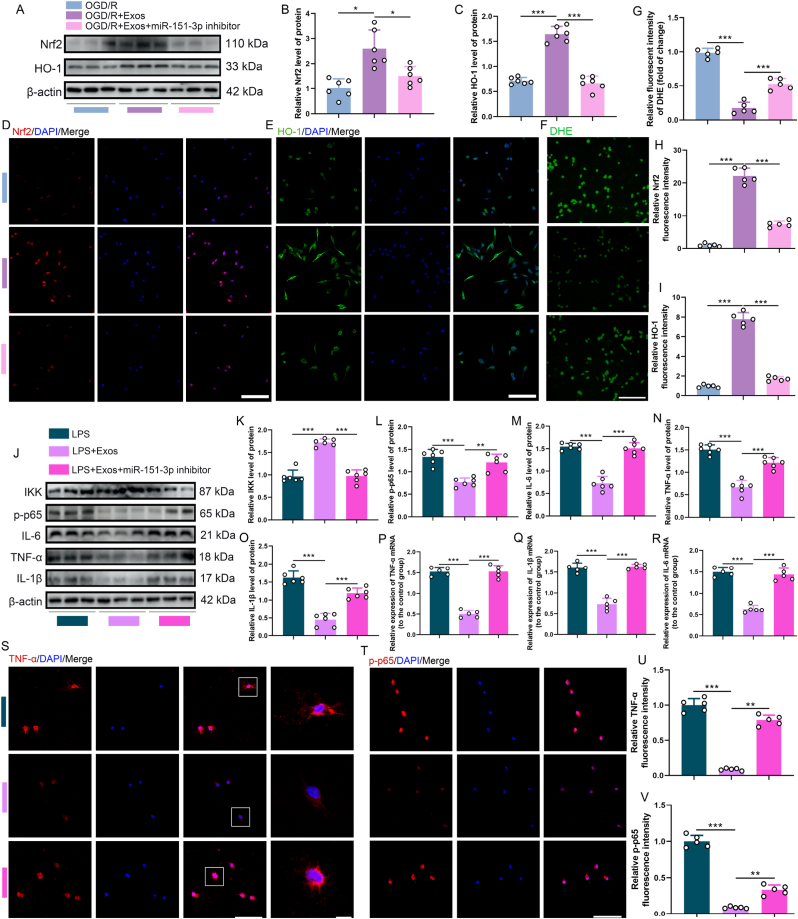
**miR-151-3p mediates antioxidant and anti-inflammatory effects of Exe-Exos. (A)** WB and **(B**–**C)** quantification of Nrf2 and HO-1 expression after OGD/R (n = 6 independent cell culture experiments). **(D)** Double-stained sections: Nrf2 (red)/DAPI (blue). **(E)** Double-stained sections: HO-1 (green)/DAPI (blue). **(F)** DHE staining of spinal cord tissues.(G) Quantification of relative DHE fluorescence intensity (fold change), (H) Nrf2 fluorescence intensity, and (I) HO‑1 fluorescence intensity (n = 5 independent cell culture experiments). **(J)** WB and **(K**–**O)** quantification of IKK, p-p65, IL-6, TNF-α, and IL-1β protein levels (n = 6 independent cell culture experiments). **(P-R**) Quantification of IL-6, TNF-α, and IL-1β mRNA levels (n = 5 independent cell culture experiments). **(S**–**T)** Double-stained sections: TNF-α (red)/DAPI (blue) and p-p65 (red)/DAPI (blue). **(U**–**V)** Quantification of relative fluorescence intensity of TNF-α (**U**) and p-p65 (**V**) (n = 5 independent cell culture experiments). Scale bar = 100 μm for all panels. *Exos*, exosomes; *Nrf2*, nuclear factor-erythroid 2-related factor 2; *HO-1*, heme oxygenase 1; *IKK*, inhibitor of kappa B kinase; *IL-6*, interleukin-6; *TNF-α*, tumor necrosis factor-α; *IL-1β*, interleukin-1β.

Given the interplay between oxidative stress and apoptosis, we next examined the Nrf2/HO-1 antioxidant pathway. Exe-Exos significantly upregulated Nrf2 and HO-1 at both protein (OGD/R + Exos *vs*. OGD/R, Nrf2 *p* < 0.05, HO-1 *p* < 0.001; OGD/R + Exos + miR-151-3p inhibitor *vs*. OGD/R + Exos, Nrf2 *p* < 0.05, HO-1 *p* < 0.001) and cellular levels (OGD/R + Exos *vs*. OGD/R, Nrf2, HO-1; OGD/R + Exos + miR-151-3p inhibitor *vs*. OGD/R + Exos, Nrf2, HO-1 [for all *p* < 0.001]), effects reversed by the miR-151-3p inhibitor (Fig. 7A–C). DHE staining further confirmed that Exe-Exos reduced ROS production, with the inhibitor abolishing this effect (OGD/R + Exos *vs.* OGD/R, *p* < 0.05; OGD/R + Exos + miR-151-3p inhibitor *vs*. OGD/R + Exos, *p* < 0.001; Fig. 7D–I).

**Fig. 8:**
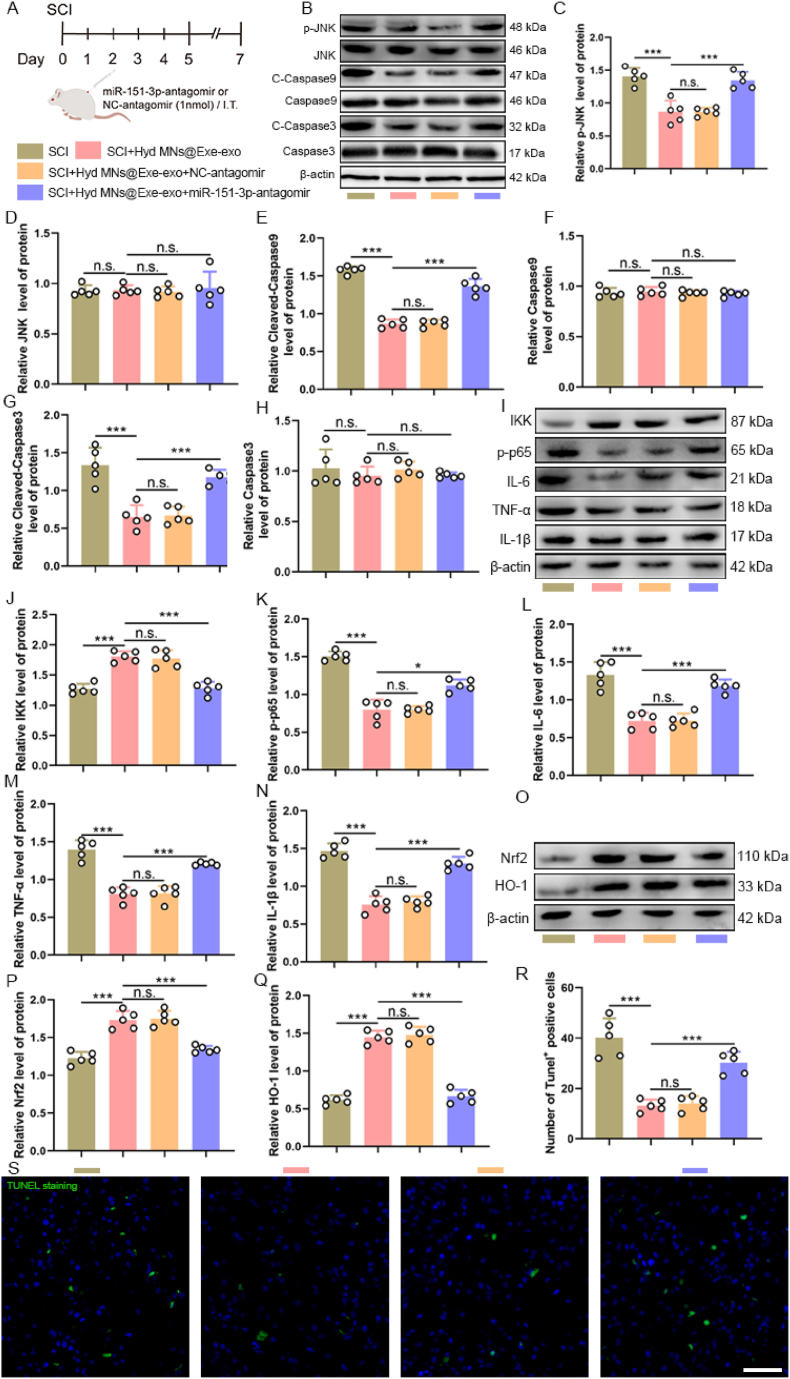
***Exo-miR-151-3p exerts anti-apoptotic, anti-inflammatory, and antioxidant effects in vivo*. (A)** Time course of intrathecal injection of miR-151-3p antagomir or NC-antagomir in rats. **(B)** WB and **(C**–**H)** quantification of p-JNK, JNK, C-Caspase9, Caspase9, C-Caspase3, and Caspase3 (n = 5 independent biological replicates). **(I)** WB and **(J**–**N)** quantification of IKK, p-p65, IL-6, TNF-α, and IL-1β (n = 5 independent biological replicates). **(O)** WB and **(P**–**Q)** quantification of Nrf2 and HO-1 (n = 5 independent biological replicates). **(R)** Quantification of TUNEL^+^ cells; (n = 5 independent biological replicates). **(S)** Representative TUNEL staining showing neuronal apoptosis at the injury epicenter. Scale bar = 20 μm. *Exos* exosomes, *JNK* c-Jun N-terminal kinase, *IKK* inhibitor of kappa B kinase, *IL-6* interleukin-6, *TNF-α* tumor necrosis factor-α, *IL-1β* interleukin-1β, *Nrf2* Nuclear factor-erythroid 2-related factor 2, *HO-1* heme Oxygenase 1, *TUNEL* TdT-mediated dUTP nick end labeling.

### 2.6 miR-151-3p mediates antioxidant effects of Exe-Exos

Apoptosis, Oxs, and inflammation form a closely intertwined “pathological triangle” that amplifies tissue damage and disease progression. To investigate the role of miR-151-3p in inflammation, we established an LPS-induced inflammatory model in microglia. In the LPS + Exos group, IKK was upregulated, while p-p65, IL-6, TNF-α, and IL-1β were downregulated compared with LPS alone. These effects were reversed by the miR-151-3p inhibitor (LPS + Exos *vs*. LPS, IKK, p-p65, IL-6, TNF-α, IL-1β; LPS + Exos + miR-151-3p inhibitor *vs*. LPS + Exos, IKK, p-p65, IL-6, TNF-α, and IL-1β [for all *p* < 0.001]; Fig. 7J–R). Immunofluorescence staining for TNF-α and p-p65 further confirmed that Exe-Exos reduced their expression and improved cell morphology, whereas the inhibitor exacerbated the inflammatory response (LPS + Exos vs. LPS: *p* < 0.001; inhibitor vs. Exos: p < 0.01; Fig. 7S–V). To further validate the gain-of-function role of miR-151-3p, PC12 cells subjected to OGD/R were transfected with a miR-151-3p mimic. WB analysis (Figure S1 A-N) revealed that, compared to cells transfected with a negative control mimic (NC-mimic), transfection with the miR-151-3p mimic significantly reduced the levels of pro-apoptotic proteins p-JNK, C-Caspase9, and C-Caspase3 (*p* < 0.001), while total JNK, Caspase9, and Caspase3 levels remained unchanged (*p* > 0.05). Furthermore, DHE staining (Figure S1 O–P) demonstrated a marked decrease in ROS levels in the miR-151-3p mimic group compared to the NC-mimic group under OGD/R conditions. These results align with the protective effects of Exe-Exos and demonstrate that miR-151-3p overexpression alone is sufficient to suppress apoptosis and activate antioxidant defense.

### 2.7 Exo-miR-151-3p exerts anti-apoptotic, anti-inflammatory, and antioxidant effects in vivo

To assess the in vivo role of miR-151-3p, rats received intrathecal injections of miR-151-3p antagomir for 5 consecutive days after SCI, and tissues were collected at 7 DPI (Fig. 8A). Exe-Exos significantly reduced the levels of p-JNK, C-Caspase9, and C-Caspase3 (SCI + Hyd MNs@Exe-Exos *vs*. SCI, *p*-JNK, C-Caspase9, C-Caspase3; SCI + Hyd MNs@Exe-Exos + miR-151-3p-antagomir *vs*. SCI + Hyd MNs@Exe-Exos, *p*-JNK, C-Caspase9, C-Caspase3 [for all *p* < 0.001, except for C-Caspase3 *p* < 0.01]) without affecting total JNK, Caspase9, or Caspase3 (*p* > 0.05). These anti-apoptotic effects were reversed by the antagomir (Fig. 8B–H). Similarly, Exe-Exos suppressed inflammatory markers (p-p65, IL-6, TNF-α, IL-1β) and upregulated IKK; all changes were reversed by the antagomir (SCI + Hyd MNs@Exe-Exos *vs*. SCI, IKK, p-p65, IL-6, TNF-α, IL-1β; SCI + Hyd MNs@Exe-Exos + miR-151-3p antagomir *vs*. SCI + Hyd MNs@Exe-Exos, IKK, p-p65, IL-6, TNF-α, IL-1β [for all *p* < 0.001, except for p-p65 *p* < 0.05]) (Fig. 8I–N). Exe-Exos also increased the antioxidant proteins Nrf2 and HO-1, an effect abolished by the antagomir (SCI + Hyd MNs@Exe-Exos *vs.* SCI, Nrf2, HO-1; SCI + Hyd MNs@Exe-Exos + miR-151-3p antagomir *vs.* SCI + Hyd MNs@Exe-Exos, Nrf2, HO-1 [for all *p* < 0.001]; Fig. 8O–Q). TUNEL staining confirmed that Exe-Exos reduced apoptosis, while the antagomir reversed this protection (SCI + Hyd MNs@Exe-Exos *vs*. SCI; SCI + Hyd MNs@Exe-Exos + miR-151-3p antagomir vs. SCI + Hyd MNs@Exe-Exos [for all *p* < 0.001]; Fig. 8R and S). No differences were observed between the Exe-Exos and Exe-Exos + NC-antagomir groups (*p* > 0.05), confirming that miR-151-3p is essential for the therapeutic effects of Exe-Exos.

### 2.8 Exosomal miR-151-3p protects mitochondrial integrity by directly targeting ROMO1

**Fig. 9:**
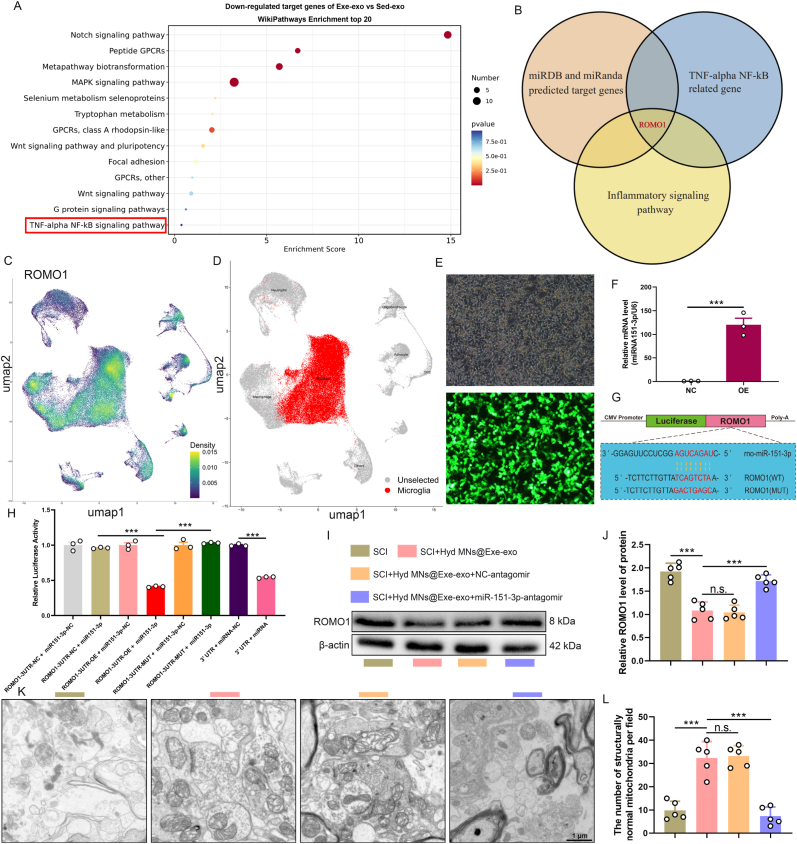
**Identification of ROMO1 as the direct target of exosomal miR-151-3p and its role in preserving mitochondrial integrity. (A)** WikiPathways enrichment analysis of downregulated target genes in Exe-Exos versus Sed-Exos (top 20 pathways). **(B)** Venn diagram intersecting miRDB- and miRanda-predicted targets, TNF-α/NF-κB-related genes, and inflammatory signaling pathway genes. Red font indicates the common intersection (ROMO1). **(C)** Distribution of ROMO1 expression across different cell types. **(D)** Visualization of microglial subpopulations. **(E**–**H)** Dual luciferase reporter assay confirming direct binding of miR-151-3p to the 3′ UTR of ROMO1 (n = 3 independent biological replicates). **(I)** WB and **(J)** quantification of ROMO1 protein expression in the four groups. **(K)** TEM images of mitochondrial structure (scale bar = 1 μm). **(L)** Quantification of structurally normal mitochondria per field (n = 5 independent biological replicates). *SCI* spinal cord injury, *Exos* exosomes, *ROMO1* reactive oxygen species modulator 1, *TEM* Transmission electron microscopy.

**Fig. 10:**
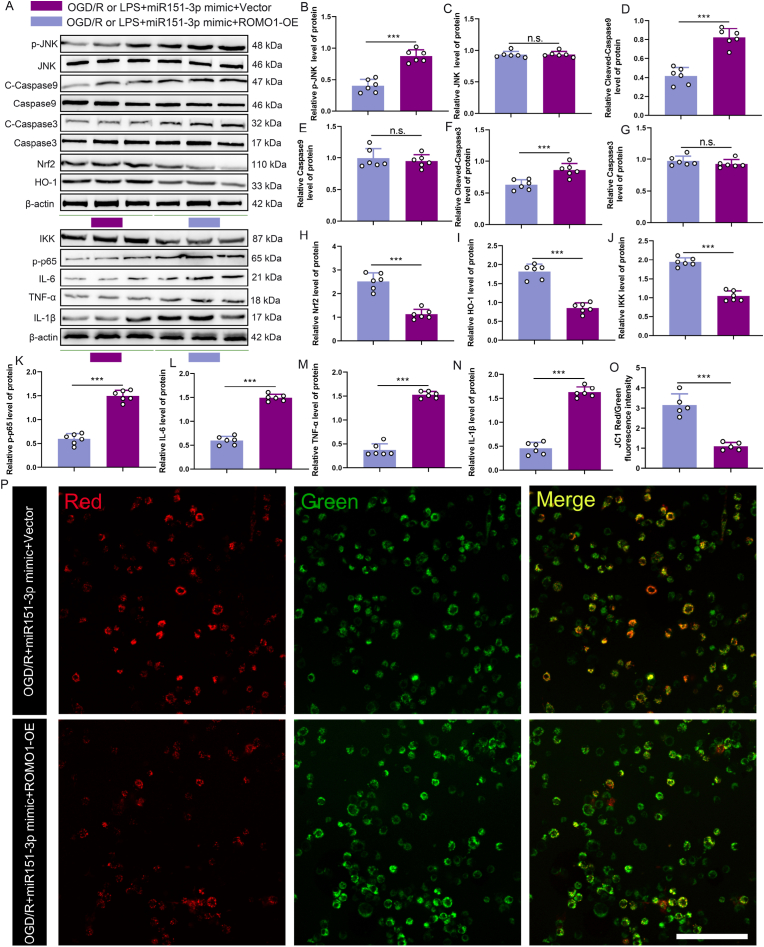
***ROMO1 overexpression reverses the protective effect of miR-151-3p mimic against OGD/R or LPS-induced injury*. (A)** WB and **(B**–**N)** quantification of p-JNK, JNK, C-Caspase9, Caspase9, C-Caspase3, Caspase3, Nrf2, HO-1, IKK, p-p65, IL-6, TNF-α, and IL-1β protein levels (n = 6 independent cell culture experiments). (**O**) Statistical analysis of the ratio of red to green fluorescence intensity for JC-1 staining (n = 5 independent biological replicates). (**P**) Representative fluorescence images of JC-1 staining for the assessment of mitochondrial membrane potential (Red fluorescence represents J-aggregates; Green fluorescence represents JC-1 monomers). Scale bar = 100 μm. *JNK* c-Jun N-terminal kinase, *IKK* inhibitor of kappa B kinase, *IL-6* interleukin-6, *TNF-α* tumor necrosis factor-α, *IL-1β* interleukin-1β, *Nrf2* Nuclear factor-erythroid 2-related factor 2, *HO-1* heme Oxygenase 1, *ROMO1* reactive oxygen species modulator 1.

To identify downstream targets of miR-151-3p, we performed WikiPathways enrichment analysis on the top 20 predicted target genes (Fig. 9A). Intersection of TNF-α, NF-κB, and inflammation-related genes with miRDB and miRanda predictions identified ROMO1 (Fig. 9B). UMAP visualization of single-cell data (GSE189070) showed ROMO1 expression predominantly in microglia (Fig. 9C–D and Figure S2A). Dual luciferase assays with a mutated 3′ UTR of ROMO1 confirmed direct binding and inhibition by miR-151-3p (Fig. 9E–H). Consistently, ROMO1 protein expression was reduced by Exe-Exos and restored by the miR-151-3p antagomir (SCI + Hyd MNs@Exe-Exos *vs*. SCI; SCI + Hyd MNs@Exe-Exos + miR-151-3p-antagomir *vs*. SCI + Hyd MNs@Exe-Exos, [for all *p* < 0.001]; Fig. 9I–J). TEM analysis revealed that Exe-Exos preserved normal mitochondrial structure in the T10 spinal cord, whereas the antagomir exacerbated disruption (SCI + Hyd MNs@Exe-Exos *vs*. SCI; SCI + Hyd MNs@Exe-Exos + miR-151-3p antagomir *vs*. SCI + Hyd MNs@Exe-Exos [for all *p* < 0.001]; Fig. 9K–L). To functionally validate ROMO1 as the key mediator, rescue experiments were performed. In OGD/R-treated PC12 cells and LPS-stimulated BV2 cells, the miR-151-3p mimic reduced p-JNK, cleaved Caspase-9/3, and upregulated Nrf2/HO-1 compared to control (Fig. 10A–N). Importantly, overexpression of ROMO1 (ROMO1-OE) largely reversed these protective effects, restoring apoptotic and oxidative stress markers to baseline levels. Consistently, JC-1 fluorescence staining (Fig. 10O–P) revealed that ROMO1 overexpression abrogated the protective effect of the miR-151-3p mimic on mitochondrial membrane potential, as indicated by a visible decrease in red-to-green fluorescence (*p* < 0.001). These findings indicate that exosomal miR-151-3p directly targets ROMO1 to preserve mitochondrial integrity, contributing to its anti-apoptotic and antioxidant effects after SCI. We acknowledge that other synergistic targets may also participate in mediating these protective effects.

## 3 Discussion

The systemic release of Exos in response to Exe is an emerging paradigm for inter-tissue communication. Seminal work has shown that Exe alters the quantity and cargo of circulating Exos, linking them to adaptive benefits in the cardiac, metabolic, and musculoskeletal systems [19,20]. Our study extends this concept to central nervous system repair. Unlike prior studies that merely described post-Exe exosomal changes, we directly harnessed Exe-Exos as a therapeutic agent and delivered them via a hydrolytically degradable MNs patch to achieve sustained local release at the SCI site—a strategy distinct from systemic administration. Moving beyond correlative observations, we identified a specific functional miRNA, miR-151-3p, and its novel mitochondrial target ROMO1, thereby elucidating a precise multi-pathway protective mechanism (anti-apoptotic, anti-inflammatory, and antioxidant mechanism) through which Exe-Exos confer neuroprotection. This triad—therapeutic application to SCI, advanced targeted delivery, and deep mechanistic dissection—defines the uniqueness of our work within the evolving fields of Exe-responsive signaling and Exos biology.

The present study aligns with and extends the growing field of Exos-based therapeutics and Exe-induced humoral signaling. Recent advances highlight Exos as versatile, cell-free delivery vehicles with superior biocompatibility and targeting potential compared with synthetic nanoparticles or stem cell transplants [[52], [53], [54]]. Our strategy of loading Exe-Exos into a hydrolytically degradable MNs patch directly addresses key translational challenges in SCI therapy: achieving sustained local drug release, minimizing systemic side effects, and bypassing the hostile injury microenvironment that often limits the efficacy of diffusible drugs or transplanted cells [45]. The GelMA-based MNs patch offers several benefits: it enables minimally invasive, targeted delivery of Exe-Exos directly to the injury epicenter, bypassing the blood-spinal cord barrier; encapsulates Exos within a biodegradable Hyd MNs that provides a protective microenvironment and ensures sustained release as the Hyd MNs degrades in vivo over approximately 1–2 weeks; and maintains a therapeutic concentration locally, reducing the need for frequent re-administration—an advantage for managing the dynamic secondary injury cascade [55,56].

We previously explored mechanisms involved in Exe rehabilitation [50,57]. However, because Exe acts at multiple levels—molecular, cellular, systemic, and psychological—its precise mechanisms remain difficult to define. Based on recent findings and our preliminary data, we propose that Exe alters the concentration of circulating Exos, that Exe-Exos contribute to functional recovery after SCI, and that specific signaling molecules within Exe-Exos drive this recovery. Exos carry proteins, nucleic acids, lipids, and metabolites from their parent cells, enabling intercellular transfer and communication that regulates diverse physiological and pathological processes [23,24,27,58]. As a cell-free approach, Exos offer high stability and low immunogenicity compared with traditional stem cell therapies, making them a focus of current research [14,[59], [60], [61]].

Traditional tail-vein injection of Exos suffers from poor targeting and subtherapeutic concentrations. In situ injection into the spinal cord also has limitations: excessive doses may aggravate tissue damage, insufficient retention time compromises efficacy, and adjacent healthy tissue can be harmed [[41], [42], [43]]. Hyd MNs offer a novel alternative, combining MNs penetrability with sustained release from Hyd MNs, and have shown promise for targeted therapy [62,63]. Various MNs platforms for Exos delivery have been developed, including dissolvable and core-shell designs [64]. In this study, we designed a Hyd MNs based on previous work to achieve targeted Exos delivery [44,65]. PKH67 membrane tracking confirmed that Exos delivered via Hyd MNs penetrate the dorsal rat spinal cord and reach the central canal, where they exert therapeutic effects.

Following SCI, dorsal spinal tissue produces numerous inflammatory cells that drive secondary tissue damage. Exe-Exos significantly improved hip, knee, and ankle mobility at 7 and 28 days post-SCI, alleviated hindlimb dragging, and promoted neural connections as evidenced by electrophysiology, explaining the restoration of motor function. Histologically, Exe-Exos reduced cavity formation and collagen deposition, enhanced nerve fiber repair, and decreased apoptosis. Notably, an analysis of the behavior of the rats using the BBB scale showed significant improvement as early as 7 days after Exe-Exos treatment, suggesting that timely suppression of the secondary injury cascade—including inflammation and oxidative stress—underlies this early benefit [[66], [67], [68]].

The identification of miR-151-3p as a key functional cargo underscores the emerging paradigm of Exe-mediated systemic communication by circulating miRNAs. Physical activity alters the miRNA profile of circulating Exos, generating Exe-responsive miRNAs that orchestrate adaptive responses in distant organs [17,69]. Recent studies have identified novel Exe-responsive miRNAs: long-term aerobic Exe enriches exosomal miR-214-3p, which enhances endothelial progenitor cell function via the PTEN-PI3K-Akt pathway and COL1A2 regulation [70]; endurance training increases exosomal miR-136-3p, derived from pancreatic islets, which enhances glucose uptake and targets NRDC in myocytes, suggesting islet–skeletal muscle crosstalk [71]; and resistance exercise induces distinct EV miRNA profiles, highlighting the diversity of cargo across training modalities [69]. Our finding that miR-151-3p is enriched in Exe-Exos and mediates multifaceted protective effects adds a novel member to this growing family. It suggests that the benefits of Exe can be partially “captured” and therapeutically redeployed via exosomal constituents, offering a promising avenue for patients with limited mobility.

Transcriptome sequencing of Exe-Exos and Sed-Exos identified 10 miRNAs upregulated in Exe-Exos, with RT-PCR confirming significant enrichment of miR-151-3p. Several other miRNAs (e.g., rno-miR-382-5p) showed extreme fold changes, which may reflect very low baseline expression in Sed controls or Exe-induced “on/off” switching of miRNA loading into Exos, making them candidates for future study. To verify the early protective effects of miR-151-3p, we conducted in vitro experiments showing that Exe-Exos-derived miR-151-3p suppressed apoptosis (JNK/Caspase), reduced inflammation (NF-κB), and promoted antioxidant responses (Nrf2/HO-1). These findings were confirmed in vivo using intrathecal injection of a miR-151-3p antagomir. Notably, total protein levels of JNK, Caspase3, and Caspase9 remained unchanged, whereas their phosphorylated or cleaved forms were significantly reduced after Exos intervention, indicating that miR-151-3p exerts functional regulation rather than simply affecting protein transcription or translation.

The link between exosomal miR-151-3p and mitochondrial ROMO1 inhibition provides a molecular bridge connecting Exe benefits to cellular homeostasis. ROMO1 is a key regulator of mitochondrial ROS production, and its overexpression amplifies Oxs and inflammation in various injury and degenerative models [72,73]. Recent studies have revealed ROMO1's dual role as both an ROS modulator and a non-selective ion channel, positioning it centrally in mitochondrial redox regulation [74]. Moreover, ROMO1 protects the mitochondrial cysteinome from harmful oxidation, and its overexpression can reverse mitochondrial oxidation and slow functional decline in aged mice [75]. Consistent with these roles in maintaining mitochondrial integrity, our JC-1 staining results from the rescue experiments confirmed that forced overexpression of ROMO1 abrogated the stabilizing effect of miR-151-3p on the mitochondrial membrane potential, driving the cells back toward mitochondrial depolarization and subsequent apoptosis. By showing that miR-151-3p directly targets ROMO1, we elucidate a key mechanism through which Exe-induced signals mitigate secondary damage in SCI. While ROMO1 is a critical downstream effector, we recognize that additional synergistic targets may also contribute to the observed protective effects. This axis---Exe → Exos-miR-151-3p ↑ → ROMO1 ↓ → (ROS ↓, inflammation ↓, apoptosis ↓) ---offers a promising therapeutic target not only for SCI but also for other conditions driven by Oxs and neuroinflammation.

To explore the mechanism of miR-151-3p, we predicted its targets using miRDB and miRanda and intersected them with TNF-α/NF-κB-related inflammatory pathways, identifying ROMO1. Analysis of GEO dataset GSE189070 revealed that ROMO1 is predominantly expressed in microglia, consistent with its role in inflammation and Oxs. ROS are physiological at low levels but damaging when excessive. ROMO1, a mitochondrial inner membrane protein, normally maintains basal ROS but can amplify Oxs and inflammation via TNF-α/NF-κB signaling [[76], [77], [78]]. Using luciferase assays with a mutated 3′ UTR, we confirmed that miR-151-3p directly binds ROMO1, and in vivo WB validated that miR-151-3p modulates ROMO1 expression.

Recent studies have demonstrated the therapeutic potential of various EV-based strategies for SCI, including mesenchymal stem cell-derived EVs, neural stem cell-derived EVs, and engineered EVs carrying specific miRNAs or proteins [53,79,80]. Comprehensive reviews highlight mechanisms such as promoting angiogenesis and axonal growth, regulating inflammation and immune responses, inhibiting apoptosis, and preserving blood-spinal cord barrier integrity [54,58]. For example, BMSC-derived Exos loaded with miR-216 promote angiogenesis and reduce neuronal apoptosis via PTEN/AKT signaling [81], and ExoPTEN—an allogeneic Exos therapy delivering siRNA against PTEN—has a seven-day therapeutic window and has received orphan drug designation for SCI [82]. Our study offers several distinctive features. First, unlike MSC- or NSC-derived EVs that require cell culture and manipulation, our Exe-Exos are harvested from the serum of exercised rats, providing a physiologically induced, non-invasively accessible EV source that aligns with “exercise mimetics” and avoids cell manufacturing challenges. Second, while others have reported beneficial miRNAs such as miR-21, miR-124, and miR-133b in SCI repair [83], we identify miR-151-3p as a previously unrecognized Exe-responsive miRNA targeting the mitochondrial protein ROMO1; this mechanism—simultaneously modulating apoptosis, Oxs, and inflammation via a single upstream target—offers a broader therapeutic profile than single-pathway interventions. Third, our Hyd MNs for sustained local delivery represents a translational advance over systemic injection or intrathecal bolus administration, minimizing off-target effects while achieving lesion-site accumulation [55]. Nevertheless, we acknowledge that the efficacy of Exe-Exos may not surpass that of optimized MSC-EVs in some contexts and that head-to-head comparisons are lacking; moreover, scalability of Exe-derived EVs from human serum remains a translational hurdle.

Our identification of exosomal miR-151-3p as a key mediator adds a new member to the therapeutic miRNA family for SCI. Contextualizing its mechanism with other well-studied miRNAs: miR-21 promotes axonal regeneration and functional recovery by targeting PTEN and activating PI3K/Akt [84]; miR-124, often delivered via stem cell-derived Exos, drives microglial polarization toward an anti-inflammatory phenotype [85]; and miR-133b enhances neurite outgrowth and angiogenesis [86]. In contrast, our mechanism centers on mitochondrial regulation. By directly targeting ROMO1 on the mitochondrial membrane, miR-151-3p concurrently suppresses the JNK/Caspase apoptotic pathway and the NF-κB inflammatory cascade while amplifying the Nrf2/HO-1 antioxidant response. This multi-faceted, integrative action---simultaneously addressing apoptosis, Oxs, and inflammation through ROMO1 as a key, but not necessarily exclusive, upstream target---distinguishes the miR-151-3p/ROMO1 axis from more pathway-specific miRNAs and likely underlies its potent neuroprotective efficacy in our model.

Clinical management of SCI remains largely supportive, with a striking lack of disease-modifying therapies [8]. Preclinical strategies such as neurotrophic factor delivery, stem cell transplantation, and biomaterial scaffolds face hurdles including instability, immune rejection, and poor functional integration [9]. Our approach harnesses the body's own adaptive response to Exe-Exos and couples it with an advanced Hyd MNs delivery platform, providing a controlled, sustained supply of protective factors directly to the lesion site and potentially creating a more permissive microenvironment for repair during the critical post-injury period [87].

Several limitations warrant acknowledgment and directly inform the next research priorities: all experiments were performed in male SD rats, so efficacy and safety in females and other species remain to be determined; only one moderate-to-severe SCI model was tested with immediate post-injury treatment, and future studies will expand to mild injuries, complete transection paradigms, and clinically relevant delayed or chronic intervention windows; while in vitro rescue experiments identified ROMO1 as a critical mediator, rigorous in vivo causal validation will be pursued using AAV-mediated, locally controlled ROMO1 modulation to avoid germline knockout–associated timing constraints and developmental compensation; key mechanistic findings in PC12 and BV2 cells will be corroborated in primary neurons and primary microglia to exclude cell line–specific artifacts; given that functional outcomes were assessed up to 28 days post-SCI, longer-term evaluations (e.g., 12 weeks) are required to establish recovery durability; finally, Hyd MNs have not yet been directly compared with alternative delivery routes, and systematic head-to-head studies will be conducted to define their relative advantages and optimize translational feasibility.

In summary, this study integrates concepts of Exe health, hemotransfusion therapy, and Exe preconditioning to establish a new SCI treatment strategy, elucidating the mechanism and therapeutic potential of Exe-Exos. Our findings demonstrate that the early anti-inflammatory and antioxidant effects of Exe-Exos are primarily mediated by miR-151-3p-dependent inhibition of ROMO1, which promotes motor recovery after SCI. To our knowledge, this is a previously unreported mechanistic insight. Accordingly, systematic validation of the efficacy and biosafety of this MNs-Exos complex in large animal models is our immediate priority. Future work will also optimize MNs release kinetics, evaluate systemic distribution and retention, and comprehensively map the “exercise exosome signature” via proteomic and miRNA profiling of Exe-Exos. Such efforts will clarify the synergistic roles of other molecules alongside miR-151-3p—an Exe-induced exosomal miRNA with defined neuroprotective functions—and provide a more holistic understanding of Exe-mediated systemic benefits.

## 4 Conclusions

In summary, this study establishes Exe-Exos as a novel, effective cell-free therapeutic strategy for SCI. Specifically: (1) localized delivery via a GelMA Hyd MNs promotes motor recovery and tissue repair; (2) these protective effects are largely mediated by miR-151-3p, which attenuates secondary injury through concurrent antiapoptotic, anti-inflammatory, and antioxidant effects; and (3) mechanistically, exosomal miR-151-3p directly targets and inhibits the mitochondrial protein ROMO1, which contributes to suppressing the JNK/Caspase apoptotic pathway, dampening NF-κB-driven inflammation, and activating the Nrf2/HO-1 antioxidant axis. We acknowledge that other synergistic targets may also play a role. These findings elucidate the precise molecular pathway by which Exe-induced neuroprotection can be harnessed and highlight that elevating miR-151-3p levels—whether through endogenous Exe or exogenous Exos delivery—represents a promising approach to improve functional recovery after SCI.

## Ethics approval and consent to participate

Experimental procedures were authorized by the Animal Research Committee of Wenzhou Medical University (reference number xmsq2023-0084) and followed the National Institutes of Health Guide for the Care and Use of Laboratory Animals.

The PC12 cell line (Resource Research Identification Code: CVCL_0481) and the BV2 cell line (Resource Research Identification Code: CVCL_0182) are cell lines from the American Type Culture Collection (ATCC).

## Funding

This research was supported by the National Natural Science Foundation of China (grants 82302856 and 82271629), the China Postdoctoral Science Foundation (grant 2024M762443), the Priority-Funded Postdoctoral Research Project of Zhejiang Province (ZJ2024133), the Postdoctoral Fellowship Program of the China Postdoctoral Science Foundation (GZC20241252), the Zhejiang Provincial Natural Science Foundation of China (LQ21H170003), and the Summit Advancement Disciplines of Zhejiang Province (Wenzhou Medical University-Pharmaceutics).

## CRediT authorship contribution statement

**Xinwang Ying:** Data curation, Formal analysis, Funding acquisition, Project administration, Software, Writing – original draft, Writing – review & editing. **Jiamen Shen:** Data curation, Formal analysis, Methodology. **Yanfang Zhao:** Formal analysis, Methodology, Software. **Tiantaixi Tu:** Formal analysis, Methodology, Software. **Yi Jiang:** Data curation, Methodology. **Bo Chen:** Data curation, Formal analysis, Methodology, Software. **Zhiyang Huang:** Data curation, Software, Supervision. **Qingfeng Xie:** Conceptualization, Data curation, Supervision, Validation. **Liuxi Chu:** Data curation, Methodology, Software. **Junqing Huang:** Formal analysis, Software. **Yanming Zuo:** Formal analysis, Resources, Software. **Ao Fang:** Investigation, Supervision, Validation. **Ping Wu:** Data curation, Formal analysis. **Qian Xu:** Data curation, Formal analysis. **Xiaokun Li:** Investigation, Project administration, Writing – original draft. **Chang Jiang:** Formal analysis, Project administration, Supervision, Writing – original draft, Writing – review & editing. **Zhouguang Wang:** Project administration, Writing – original draft, Writing – review & editing.

## Declaration of competing interest

The authors declare that they have no known competing financial interests or personal relationships that could have appeared to influence the work reported in this paper.

## Data Availability

The raw microRNA sequencing data generated in this study have been deposited in the NCBI GEO database under accession number GSE317223. Additional data supporting the findings of this study are available from the corresponding author upon reasonable request.

## Abbreviations

BBB, Basso–Beattie–Bresnahan; BCA, bicinchoninic acid; DAPI, 4,6-diamidino-2-phenylindole; DHE, dihydroethidium; DPI, days postinjury; Exe, exercise; Exos, exosomes; FBS, fetal bovine serum; GelMA, gelatin methacrylate; H&E, hematoxylin–eosin; HO-1, heme oxygenase 1; Hyd MNs, hydrogel microneedles; IKK, inhibitor of kappa B kinase; IL, interleukin; JNK, C-jun N-terminal kinase; MAA, methacrylic anhydride; MEP, motor evoked potential; miR, microRNA; min, minutes; Nrf2, nuclear factor-erythroid 2-related factor 2; NTA, nanoparticle tracking analysis; OGD/R, oxygen-glucose deprivation/reperfusion; OPCs, oligodendrocyte precursor cells; Oxs, oxidative stress; PBS, phosphate-buffered saline; ROMO1, reactive oxygen species modulator 1; ROS, reactive oxygen species; SC, spinal cord; SCI, spinal cord injury; Sed, sedentary; TEM, transmission electron microscopy; TNF-α, tumor necrosis factor-α; TUNEL, TdT-mediated dUTP nick end labeling; UMAP, uniform manifold approximation and projection.
