# Supplementary material for: Exercise-derived exosomal miR-151-3p: An innovative anti-inflammatory and antioxidant therapeutic for spinal cord injury
Source: Bioact Mater. 2026 Jun 13;65:535–55. doi: 10.1016/j.bioactmat.2026.06.009 (PMC13276775; doi:10.1016/j.bioactmat.2026.06.009)
Supplement: Multimedia component 2 [file mmc2.docx]

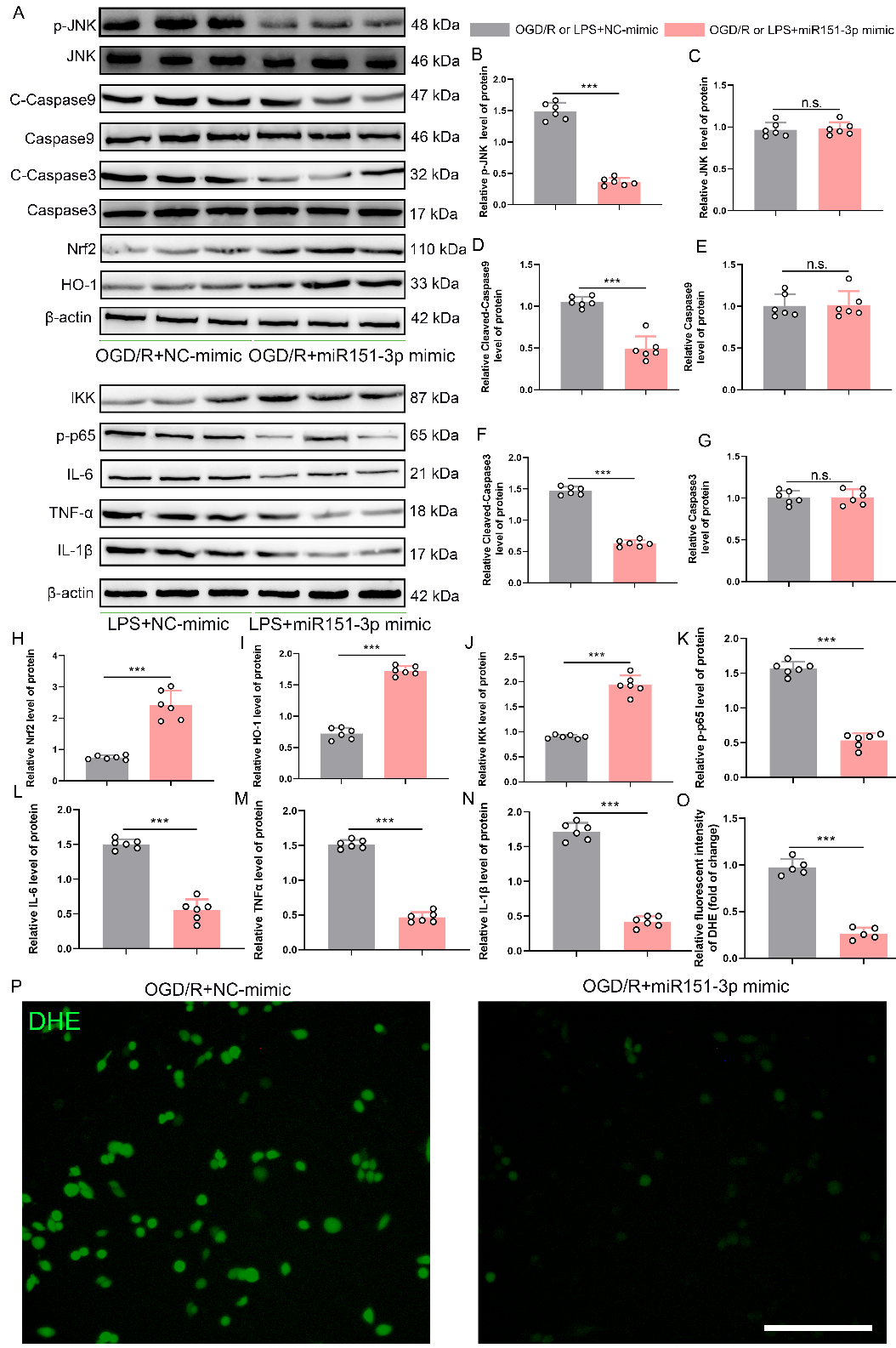


**Figure S1. *miR-151-3p mimic suppresses the NF‑κB inflammatory pathway and reduces ROS production.*** (**A**) WB and **(B–N)** quantification of *p*-JNK, JNK, C-Caspase9, Caspase9, C-Caspase3, Caspase3, Nrf2, HO-1, IKK, p-p65, IL-6, TNF-α, and IL-1β protein levels; (n = 6 independent cell culture experiments). (**O**) Quantification and (**P**) DHE staining for ROS in the indicated groups. Scale bar = 100 µm. (n = 5 independent cell culture experiments). *DHE* dihydroethidium, *ROS* reactive oxygen species, *HO-1* heme oxygenase 1, *IKK* inhibitor of kappa B kinase, *IL* interleukin, *JNK* C-jun N-terminal kinase, *Nrf2* nuclear factor-erythroid 2-related factor 2.


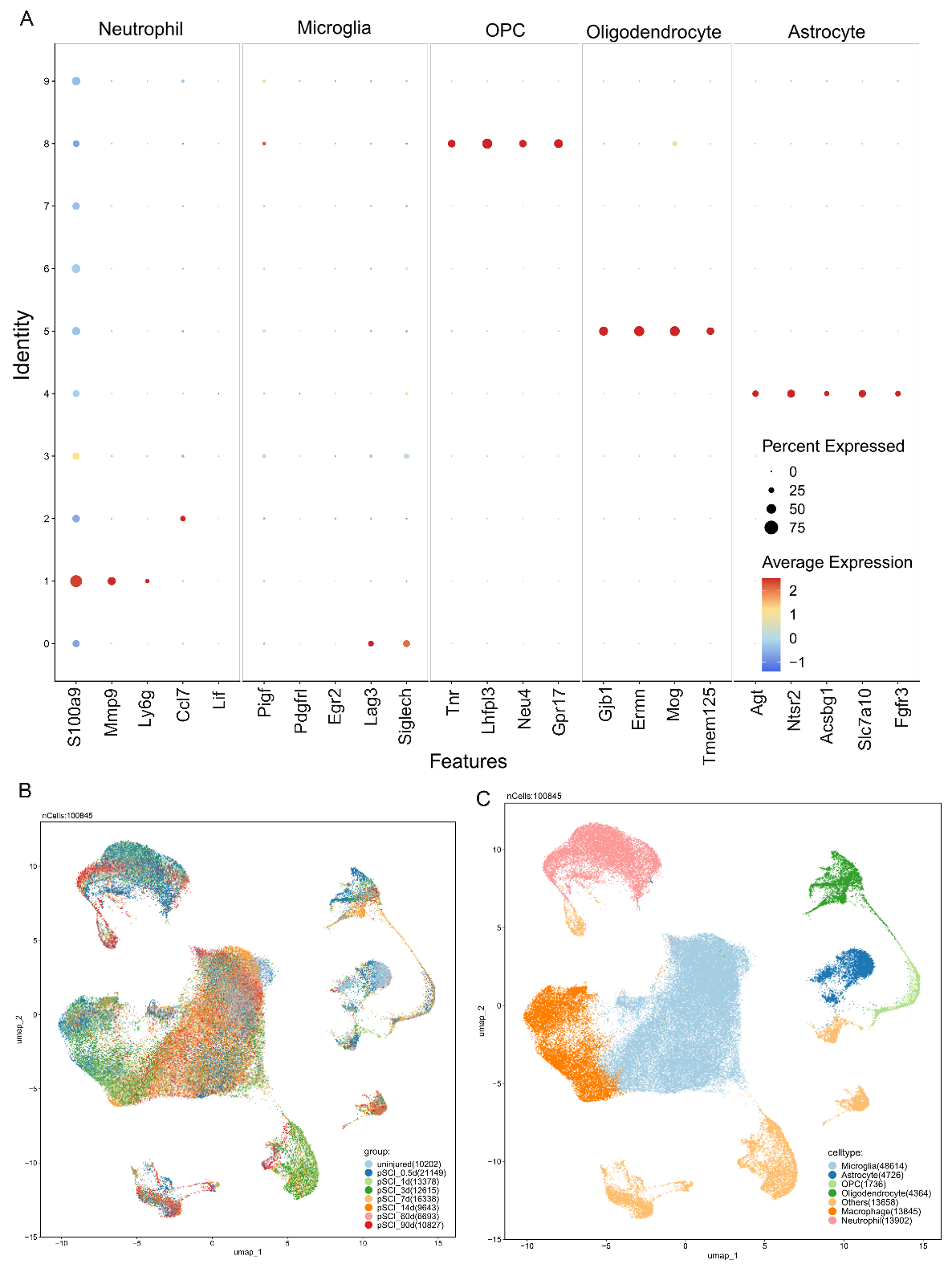


**Figure S2. *Identification of ROMO1 as the target gene of Exo‑miR‑151‑3p by single‑cell transcriptomics.* (A)** Cell type annotation of the GEO single‑cell dataset GSE189070. **(B)** UMAP plot showing sample distribution across single‑cell datasets. **(C)** Cell types based on annotation. *OPC* oligodendrocyte precursor cells, *UMAP* uniform manifold approximation and projection.
